# Supplementary material for: Dietary Macronutrient Intake and Cardiovascular Disease Risk and Mortality: A Systematic Review and Dose-Response Meta-Analysis of Prospective Cohort Studies
Source: Nutrients. 2024 Jan 2;16(1):152. doi: 10.3390/nu16010152 (PMC10780780; doi:10.3390/nu16010152)
Supplement: Supplementary file 1 [file nutrients-16-00152-s001.zip › nutrients-2751587-supplementary.pdf]

Online Supplemental Material

Re: Yibin Ma et al. Dietary Macronutrient Intake and Cardiovascular Disease Risk and Mortality: A Systematic Review and Dose-Response Meta-analysis of Prospective Cohort Studies

Table of Contents

Supplemental Tables **2**

Table S1: PRISMA 2020 Checklist 2

Table S2: MOOSE (Meta-analyses of Observational Studies in Epidemiology) Checklist. 5

Table S3: PECOTS framework of the search strategy 7

Table S4: Characteristics of included studies for association between macronutrients intake and CVD events in adults aged 19 or older. 8

Table S5: Characteristics of included studies for association between macronutrients intake and all cause mortality and cause-specific mortality in adults aged 19 or older. 11

Table S6: Variety of Macronutrients intake assessment and outcome methods 16

Table S7: Confounding variables of included prospective cohort studies. 25

Table S8: Newcastle-Ottawa Scale (NOS) scores of included prospective cohort studies 32

Table S9: gender subgroup-analysis of dietary macronutrient intake and on mortality or CVD events 40

Table S10: religion subgroup-analysis of dietary macronutrient intake and on mortality or CVD events 42

Table S11: Begg’s test and Egger's test for publication bias among studies 44

Supplemental Tables

Table S1. PRISMA or MOOSE Health Research Reporting Checklist.

Section and Topic	Item #	Checklist item	Location where item is reported
**TITLE**			
Title	1	Identify the report as a systematic review.	1
**ABSTRACT**			
Abstract	2	See the PRISMA 2020 for Abstracts checklist.	1
**INTRODUCTION**			
Rationale	3	Describe the rationale for the review in the context of existing knowledge.	2
Objectives	4	Provide an explicit statement of the objective(s) or question(s) the review addresses.	2
**METHODS**			
Eligibility criteria	5	Specify the inclusion and exclusion criteria for the review and how studies were grouped for the syntheses.	3, Supplemental Table S3
Information sources	6	Specify all databases, registers, websites, organisations, reference lists and other sources searched or consulted to identify studies. Specify the date when each source was last searched or consulted.	3
Search strategy	7	Present the full search strategies for all databases, registers and websites, including any filters and limits used.	
Selection process	8	Specify the methods used to decide whether a study met the inclusion criteria of the review, including how many reviewers screened each record and each report retrieved, whether they worked independently, and if applicable, details of automation tools used in the process.	Figure 1
Data collection process	9	Specify the methods used to collect data from reports, including how many reviewers collected data from each report, whether they worked independently, any processes for obtaining or confirming data from study investigators, and if applicable, details of automation tools used in the process.	3
Data items	10a	List and define all outcomes for which data were sought. Specify whether all results that were compatible with each outcome domain in each study were sought (e.g. for all measures, time points, analyses), and if not, the methods used to decide which results to collect.	3
	10b	List and define all other variables for which data were sought (e.g. participant and intervention characteristics, funding sources). Describe any assumptions made about any missing or unclear information.	3 Supplemental Tables S4-S5
Study risk of bias assessment	11	Specify the methods used to assess risk of bias in the included studies, including details of the tool(s) used, how many reviewers assessed each study and whether they worked independently, and if applicable, details of automation tools used in the process.	3
Effect measures	12	Specify for each outcome the effect measure(s) (e.g. risk ratio, mean difference) used in the synthesis or presentation of results.	3
Synthesis methods	13a	Describe the processes used to decide which studies were eligible for each synthesis (e.g. tabulating the study intervention characteristics and comparing against the planned groups for each synthesis (item #5)).	4, Supplemental Tables S4-S5
	13b	Describe any methods required to prepare the data for presentation or synthesis, such as handling of missing summary statistics, or data conversions.	4

Section and Topic	Item #	Checklist item	Location where item is reported
	13c	Describe any methods used to tabulate or visually display results of individual studies and syntheses.	3-4, Supplemental Tables S4-S5
	13d	Describe any methods used to synthesize results and provide a rationale for the choice(s). If meta-analysis was performed, describe the model(s), method(s) to identify the presence and extent of statistical heterogeneity, and software package(s) used.	3-4
	13e	Describe any methods used to explore possible causes of heterogeneity among study results (e.g. subgroup analysis, meta-regression).	3-4
	13f	Describe any sensitivity analyses conducted to assess robustness of the synthesized results.	3-4
Reporting bias assessment	14	Describe any methods used to assess risk of bias due to missing results in a synthesis (arising from reporting biases).	3-4
Certainty assessment	15	Describe any methods used to assess certainty (or confidence) in the body of evidence for an outcome.	3-4
**RESULTS**			
Study selection	16a	Describe the results of the search and selection process, from the number of records identified in the search to the number of studies included in the review, ideally using a flow diagram.	Figure 1
	16b	Cite studies that might appear to meet the inclusion criteria, but which were excluded, and explain why they were excluded.	4-5
Study characteristics	17	Cite each included study and present its characteristics.	Supplemental Tables S4-S5
Risk of bias in studies	18	Present assessments of risk of bias for each included study.	Supplemental Table S8
Results of individual studies	19	For all outcomes, present, for each study: (a) summary statistics for each group (where appropriate) and (b) an effect estimate and its precision (e.g. confidence/credible interval), ideally using structured tables or plots.	Figures 2-7, Supplemental Figures 1-38
Results of syntheses	20a	For each synthesis, briefly summarise the characteristics and risk of bias among contributing studies.	12-13
	20b	Present results of all statistical syntheses conducted. If meta-analysis was done, present for each the summary estimate and its precision (e.g. confidence/credible interval) and measures of statistical heterogeneity. If comparing groups, describe the direction of the effect.	7-13, Figures 2-7, Supplemental Figures 1-38
	20c	Present results of all investigations of possible causes of heterogeneity among study results.	15-16, Supplemental Table S11
	20d	Present results of all sensitivity analyses conducted to assess the robustness of the synthesized results.	15-16, Supplemental Table S11
Reporting biases	21	Present assessments of risk of bias due to missing results (arising from reporting biases) for each synthesis assessed.	15-16, Supplemental Table S11
Certainty of evidence	22	Present assessments of certainty (or confidence) in the body of evidence for each outcome assessed.	15-16
**DISCUSSION**			

Section and Topic	Item #	Checklist item	Location where item is reported
Discussion	23a	Provide a general interpretation of the results in the context of other evidence.	16-17
	23b	Discuss any limitations of the evidence included in the review.	20-21
	23c	Discuss any limitations of the review processes used.	20-21
	23d	Discuss implications of the results for practice, policy, and future research.	21
**OTHER INFORMATION**			
Registration and protocol	24a	Provide registration information for the review, including register name and registration number, or state that the review was not registered.	Abstract, 1
	24b	Indicate where the review protocol can be accessed, or state that a protocol was not prepared.	Abstract, 6
	24c	Describe and explain any amendments to information provided at registration or in the protocol.	NA
Support	25	Describe sources of financial or non-financial support for the review, and the role of the funders or sponsors in the review.	21
Competing interests	26	Declare any competing interests of review authors.	21
Availability of data, code and other materials	27	Report which of the following are publicly available and where they can be found: template data collection forms; data extracted from included studies; data used for all analyses; analytic code; any other materials used in the review.	21-22

From: Page MJ, McKenzie JE, Bossuyt PM, Boutron I, Hoffmann TC, Mulrow CD, et al. The PRISMA 2020 statement: an updated guideline for reporting systematic reviews. BMJ 2021;372:n71. doi: 10.1136/bmj.n71.

Table S2. MOOSE (Meta-analyses of Observational Studies in Epidemiology) Checklist[a].

Reporting Criteria	Reported (Yes/No)	Reported on Page No.
Reporting of Background		
Problem definition	Yes	1-2
Description of Study Outcome(s)	Yes	1-2
Type of exposure or intervention used	Yes	1-3
Study population	Yes	1-3
Reporting of Search Strategy		
Qualifications of searchers (eg, librarians and investigators)	Yes	1
Search strategy, including time period included in the synthesis and keywords	Yes	2-3, Supplemental Table S3
Effort to include all available studies, including contact with authors	Yes	3
Databases and registries searched	Yes	3
Search software used, name and version, including special features used (eg, explosion)	Yes	4
Use of hand searching (eg, reference lists of obtained articles)	Yes	3-4, Figure 1, Supplemental Table S3
List of citations located and those excluded, including justification	Yes	Figure 1
Method for addressing articles published in languages other than English	Yes	3
Method of handling abstracts and unpublished studies	Yes	3, Supplemental Table S3
Description of any contact with authors	Yes	3, 4
Reporting of Methods		
Description of relevance or appropriateness of studies assembled for assessing the hypothesis to be tested	Yes	3
Rationale for the selection and coding of data (eg., sound clinical principles or convenience)	Yes	3
Documentation of how data were classified and coded (eg., multiple raters, blinding, and interrater reliability)	Yes	3
Assessment of confounding (eg., comparability of cases and controls in studies were appropriate)	Yes	3
Reporting Criteria		
Assessment of study quality, including blinding of quality assessors; stratification or regression on possible predictors of study results	Yes	3

Assessment of heterogeneity	Yes	3
Description of statistical methods (eg., complete description of fixed or random effects models, justification of whether the chosen models account for predictors of study results, dose-response models, or cumulative meta-analysis) in sufficient detail to be replicated	Yes	3,4
Provision of appropriate tables and graphics	Yes	Table 1, Figures 1-7, Supplemental Material
Reporting of Results		
Table giving descriptive information for each study included	Yes	Table 1, Supplemental Tables S3-S4
Results of sensitivity testing (eg., subgroup analysis)	Yes	15, 16 Supplemental Tables S9-S10
Indication of statistical uncertainty of findings	Yes	15,16 Supplemental Table S11
Reporting of Discussion		
Quantitative assessment of bias (eg., publication bias)	Yes	21
Justification for exclusion (eg., exclusion of non-English-language citations)	Yes	NA
Assessment of quality of included studies	Yes	7, Supplemental Table S 8
Reporting of Conclusions		
Consideration of alternative explanations for observed results	Yes	16-21
Generalization of the conclusions (ie, appropriate for the data presented and within the domain of the literature review)	Yes	16-21
Guidelines for future research	Yes	21
Disclosure of funding source	Yes	21

NA = Not applicable. [a]StroupDF, Berlin JA, Morton SC, Olkin I, WilliamsonGD, Rennie D, MoherD, Becker BJ, Sipe TA, Thacker SB. Meta-analysis of observational studies in epidemiology: aproposal for reporting. Meta-analysis Of Observational Studies in Epidemiology (MOOSE) group. JAMA. 2000 Apr 19;283(15):2008-12. doi: 10.1001/jama.283.15.2008. PMID: 10789670.

Table S3. PECOTS framework of the search strategy.

**PECOTS framework[a] defined in the present systematic review and meta-analysis**					
**Participants**	**Exposure**	**Comparators**	**Outcomes**	**Time/ Duration**	**Setting/ Study Design**
Adults (≥ 18 years) of any sex, gender, and ethnicity, and free of CVD at baseline (for analysis of CVD-related incidence) otherwise of any health status.	Higher variety of protein and/or fat and/or carbohydrate consumption in the diet.	Lower variety of protein and/or fat and/or carbohydrate consumption in the diet.	*Mortality:*<br>All-cause<br>CVD-related<br>cancer-related<br><br>*CVD incidence:*<br>Overall CVD<br>CHD<br>Stroke	prospective cohort (at least 1 year in duration).	

Abbreviations: CHD, coronary heart disease; CVD, cardiovascular disease; [a]MoherD, ShamseerL, Clarke M, GhersiD, LiberatiA, Petticrew M, Shekelle P, Stewart LA and PRISMA-P Group. Preferred reporting items for systematic review and meta-analysis protocols (PRISMA-P) 2015 statement. Systematic Reviews 2015; 4:1. https://doi.org/10.1186/2046-4053-4-1.

Table S4. Characteristics of included studies for association between macronutrients intake and CVD events in adults aged 19 or older.

Author, publication year	Study cohort/ populations	Country	baseline age (years, mean or range)	female (%)	Follow-up (years)	number of participants	Exposure	Quantity (Highest vs Lowest Intake)	Number of CVD events			NOS score
									CVD	CHD	stroke	
**McGee et al., 1984[91]**	The Honolulu Heart Program	Japan	≥45	0	10	7544	SFA	Q5 vs Q1	NA	1177	492	8
**Fehily et al., 1993[93]**	the Caerphilly Study	UK	45-95	0	5	2432	SFA	Q3 vs Q1	NA	21	NA	7
**Goldbourt et al., 1993[34]**	NA	Israel	≥40	0	23	10059	SFA	Q4 vs Q1	NA	1070	362	6
**Ascherio et al., 1995[96]**	HPFS	US	40-75	0	6	44895	n-3 fatty acids	Q5 vs Q1	NA	1543	NA	8
**Ascherio et al., 1996[35]**	HPFS	US	40-75	0	6	43757	total fat, SFA	Q5 vs Q1	NA	737	NA	8
**Gillman et al., 1997[97]**	The Framingham Heart Study	US	40-65	0	20	832	total fat, SFA, MUFA, PUFA	15% energy	NA	NA	61	8
**Hu et al.,1997[98]**	NHS	US	34-59	100	15	80082	total fat, SFA, MUFA, PUFA, animal fat, plant fat	Q5 vs Q1	NA	658	NA	8
**Pietinen et al., 1997[38]**	The Alpha Tocopherol, Beta Carotene Cancer Prevention Study	Finland	50-69	0	6.1	21930	SFA, MUFA ,PUFA	Q5 vs Q1	NA	1399	NA	7
**Seino et al., 1997[99]**	The Shibata Study	Japan	≥40	58.2	15.5	2283	total fat,SFA,MUFA,PUFA	Q4 vs Q1	NA	NA	75	7
**Hu et al.,1999[101]**	NHS	US	34-59	100	15	80082	SFA subgroup	Q5 vs Q1	NA	939	NA	8
**Hu et al.,1999[102]**	NHS	US	NA	100	15	80082	total protein, animal protein, plant protein	Q5 vs Q1	NA	658	NA	8
**Liu et al.,2000[104]**	NHS	US	38-63	100	10	75521	carbohydrate	Q5 vs Q1	761	NA	NA	8
**Iso et al.,2001[106]**	NHS	US	NA	100	14	85764	animal protein, plant protein, total fat, SFA, MUFA, PUFA	Q5 vs Q1	NA	NA	690	8
**He et al.,2003[107]**	HPFS	US	40-75	0	14	43743	total fat, SFA, MUFA, PUFA, animal fat, plant fat	Q5 vs Q1	NA	NA	725	8
**Hu et al.,2003[108]**	NHS	US	30-55	100	16	5130	ω-3 Fatty Acids	Q5 vs Q1	NA	362	NA	8
**Iso et al.,2003[109]**	NA	Japan	40-69	52.5	14.3	4775	total fat, SFA, MUFA, PUFA	Q4 vs Q1	NA	NA	186	8
**Zhang et al., 2003[110]**	SWHS	China	40-70	100	2.5	64915	total soy protein	Q4 vs Q1	NA	62	NA	7
**Jakobsen et al.,2004[112]**	the Research Centre for Prevention and Health	Denmark	30-71	50.2	16	3686	total fat, SFA, MUFA, PUFA	intake of 5% higher level of energy from dietary fat	NA	326	NA	8
**Tanasescu et al.,2004[113]**	NHS	US	40-55	100	19	5672	total fat, SFA, MUFA, PUFA, animal fat, plant fat	Q5 vs Q1	619	451	168	8
**Oh et al.,2005[115]**	NHS	US	30-55	100	18	78779	carbohydrate	Q5 vs Q1	NA	NA	1020	8
**Oh et al.,2005[116]**	NHS	US	30-55	100	18	78779	total fat, SFA, MUFA, PUFA	Q5 vs Q1	NA	NA	1020	8
**Trichopoulou et al. 2006[117]**	Greek-EPIC Cohort	Greece	NA	48	4.5	1013	SFA, MUFA, PUFA		80	46	19	9

Supplemental Table S4 | Continued

author, publication year	Study cohort/ populations	Country	baseline age (years, mean or range)	female (%)	Follow-up (years)	number of participants	Exposure	Quantity (Highest vs Lowest Intake)	Number of CVD events			NOS score
									CVD	CHD	stroke	
**Xu et al.,2006[46]**	the Strong Heart Study	US<br>US	47–59<br>60–79	61.1<br>68.3	7.2±2.3<br>7.2±2.3	1659<br>1279	total fat, SFA, MUFA, PUFA	Q4 vs Q1	NA<br>NA	185<br>218	NA<br>NA	8
**Leosdottir et al., 2007[118]**	The Malmö Diet and Cancer Study	Sweden	57	69.0	8.4	28098	total fat, SFA, MUFA, PUFA	Q4 vs Q1	1556	908	648	6
**Boden-Albala et al.,2009[120]**	the Northern Manhattan Study	US	69	63	5.5	3183	total fat	Q5 vs Q1	NA	NA	142	7
**Preis et al., 2010[123]**	HPFS	US	40-75	0	18	51529	total protein	Q5 vs Q1	NA	2959	NA	8
**Yamagishi et al.,2010[124]**	JACC	Japan	40-79	60.6	14.1	58453	SFA	Q5 vs Q1	NA	420	321	9
**Atkinson et al.,2011[125]**	the Caerphilly cohort	US	45-59	0	18	2710	total fat, SFA	Q5 vs Q1	NA	NA	225	7
**Houston et al.,2011[126]**	the Health ABC Study	US	66,48-79	44.4	9	1941	total fat, SFA, MUFA, PUFA	Q3 vs Q1	203	NA	NA	8
**de Oliveira et al.,2012[128]**	United States MESA Cohort	US	45-84	50	10	5209	SFA	For each 5% of energy	316	231	NA	8
**Dilis et al.,2012[47]**	The EPIC cohort	Greek	20-86	59.3	10	23929	total fat, SFA, MUFA, PUFA	T3 vs T1	NA	426	NA	8
**Larsson et al.,2012[129]**	the Swedish Mammography Cohort	Sweden	49-83	100	10.4	34670	total protein, animal protein, plant protein	Q5 vs Q1	NA	NA	1680	8
**Larsson et al.,2012[61]**	the Swedish Mammography Cohort	Sweden	49-83	100	10.4	34670	total fat, SFA, MUFA, PUFA	Q5 vs Q1	NA	NA	1680	8
**Wallstrom et al.,2012[131]**	Swedish population-based Malmö Diet and Cancer cohort	Sweden	44-73	0<br>100	13.5<br>13.5	8139<br>12535	total protein, total fat, SFA, MUFA, PUFA, carbohydrate	Q5 vs Q1<br>Q5 vs Q1	1089<br>NA	688<br>333	401<br>354	8
**Yaemsiri et al.,2012[132]**	the WHI-OS	US	63.5±7.3, 50-79	100	7.6	87025	total fat, SFA, MUFA, PUFA	Q5 vs Q1	NA	NA	1049	7
**Simila et al.,2013[134]**	ATBC	Finland	50-69	0	19	21955	Carbohydrate	Q5 vs Q1	NA	4379	NA	7
**Yamagishi et al.,2013[135]**	JPHC	Japan	45-64,45-74	53.5	11.1	81931	SFA	Q5 vs Q1	NA	NA	3192	8
**Yu et al.,2013[136]**	China Shanghai study	China	40-74	55.3	F:9.8, M:5.4	117366	Carbohydrate	Q4 vs Q1	NA	309	NA	8
**Haring et al.,2014[138]**	ARIC	US	45-64	55.8	22	12066	total protein, animal protein, plant protein	Q5 vs Q1	1147	NA	NA	7
**dos Santos et al.,2014[137]**	the Diabetes research outpatient clinic at Hospital de Clínicas de Porto Alegre	Brazil	59±10	54.2	4.6	227	PUFA	Q4 vs Q1	36	NA	NA	6
**Virtanen et al.,2014[142]**	KIHD	Finland	42-60	0	21.4	1981	total fat, SFA, MUFA, PUFA	Q4 vs Q1	NA	565	NA	7
**Chiuve et al.,2015[144]**	WHS	US	≥45	100	20	33665	total fat, SFA, MUFA, PUFA	Q5 vs Q1	NA	1441		8
**Guasch-Ferré et al.,2015[62]**	the PREDIMED study	Spain	67±6	57.5	6	7038	total fat, SFA, MUFA, PUFA	Q5 vs Q1	336	NA	NA	9
**Li et al.,2015[145]**	NHS<br>HPFS	US<br>US	30-55<br>40-75	100<br>0	30<br>24	84628<br>42908	total fat, SFA, MUFA, PUFA, carbohydrate	Q5 vs Q1<br>Q5 vs Q1	NA	7667	NA	8
**Puaschitz et al.,2015[147]**	WENBIT	Norway	61.7	19	4.8	2412	SFA	Q4 vs Q1	NA	292	NA	7

Supplemental Table S4 | Continued

author，publication year	Study cohort/ populations	Country	baseline age (years, mean or range)	female (%)	Follow-up (years)	number of participants	Exposure	Quantity (Highest vs Lowest Intake)	Number of CVD events			NOS score
									CVD	CHD	stroke	
Chen et al.,2016[148]	HPFS	US	40-75	0		51529	total fat	Q5 vs Q1	5825	4422	1403	8
	NHS	US	30-55	100		92468	total fat	Q5 vs Q1	8018	4055	3963	
	NHS II	US	25-42	100		97604	total fat	Q5 vs Q1	972	497	475	
Praagman et al.,2016[152]	The EPIC-NL cohort	Holland	49.3	75	12.2	35597	SFA	Q5 vs Q1	NA	1807	NA	7
Xu et al.,2016[154]	ULSAM	Sweden	71.0 ± 0.6	0	9.1	390	total protein	per SD 8.37 g/day higher	164	NA	NA	7
Zong et al., 2016[155]	NHS<br>HPFS	US	50.2<br>53.1	63.2	25.8<br>21.2	115782	SFA subgroup	Q5 vs Q1	NA	7035	NA	8
Dehghan et al., 2017[13]	PURE	18 countries*	50.29 ±9.92，35-70	58.7	7.4	135335	total protein, total fat, SFA, MUFA, PUFA, carbohydrate	Q5 vs Q1	4787	2143	2234	8
AlEssa et al., 2018[159]	NHS	US	30-55	100	28	75,020	Carbohydrate	Q5 vs Q1	NA	3267	NA	8
	HPFS	US	40-75	0	26	42,865	Carbohydrate	Q5 vs Q1	NA	4053	NA	
Okada et al., 2019[168]	CIRCS	Japan	40-59	48.8	24.6	3248	total protein, SFA, MUFA, PUFA, carbohydrate	Q4 vs Q1	NA	NA	230	7
Rhee et al., 2017[157]	WHS	US	45+	100	21	38392	Marine omega-3 fatty acids	Q5 vs Q1	1941	NA	987	7
Mirmiran et al., 2020[176]	TLGS	Iran	38.5 ± 13.3（19-70）	56.5	6.7	2369	total fat	T3 vs T1	79	NA	NA	8
Sadeghi et al., 2021[182]	ICS	Iran	≥35	51.3	11.5	5432	animal fat	Q4 vs Q1	751	401	157	8

HPFS: The Health Professionals Follow-up Study; NHS: The Nurses' Health Study cohort; SWHS: The Shanghai Women's Health Study;EPIC: European Prospective Investigation into Cancer and Nutrition Study; ATBC: The Alpha-Tocopherol, Beta-Carotene Cancer Prevention Study; ARIC: The Atherosclerosis Risk in Communities Study; KIHD: the population-based Kuopio Ischemic Heart Disease Risk Factor Study; WHS: the Women's Health Study; WENBIT: the Western Norway B-Vitamin Intervention Trial; ULSAM: the Uppsala Longitudinal Study of Adult Men; PURE: The Prospective Urban Rural Epidemiology study; CIRCS: The Circulatory Risk in Communities Study; ICS: Isfahan Cohort Study; Health ABC: the Health, Aging and Body Composition Health ABC study; JACC: the Japan Collaborative Cohort Study; JPHC: Japan Public Health Center–based Prospective Cohort Study; PREDIMED : PREvención con DIeta MEDiterránea cohort; The EPIC-NL cohort: the European Prospective Investigation into Cancer and Nutrition–Netherlands cohort. TLGS: the Tehran Lipid and Glucose Prospective Study. *from 18 countries, three high-income (Canada, Sweden, and United Arab Emirates), 11 middle-income (Argentina, Brazil, Chile, China, Colombia, Iran, Malaysia, occupied Palestinian territory, Poland, South Africa, and Turkey) and four low-income countries (Bangladesh, India, Pakistan, and Zimbabwe).

Table S5. Characteristics of included studies for association between macronutrients intake and all cause mortality and cause-specific mortality in adults aged 19 or older.

author, publication year	Study cohort/ populations	Country	baseline age (years, mean or range)	female (%)	Follow-up (years)	number of participants	Exposure	Quantity (Highest vs Lowest Intake)	Number of death			NOS score
									All deaths	CVD deaths	Cancer deaths	
**Posner et al., 1991[92]**	The Framingham Study	US	45-55,56-65	0	16	813	total fat, SFA, MUFA, PUFA	**NA**	NA	NA	NA	8
**Rohan et al., 1993[94]**	SACCR	Australia	20-74	100	5.5	412	total protein,total fat, SFA, MUFA, PUFA, carbohydrates	Q5 vs Q1	NA	NA	112	8
**Dwyer et al., 1994[95]**	NHANES I	US	65-74	47.9	14.5	2572	total protein	additional 15 g of protein per day	119	NA	NA	9
**Esrey et al., 1996[36]**	NA	US	30-59,60-79	48.2	12.4	4546	total protein,total fat, SFA, MUFA, PUFA, carbohydrates	per 1% increase	NA	92	NA	7
**Mann et al., 1997[37]**	vegetarians, meat eaters	UK	33,16-79	62	13.3	10802	total animal fat, saturated animal fat	Q3 vs Q1	392	64	NA	8
**Pietinen et al.,1997[38]**	The Alpha Tocopherol, Beta Carotene Cancer Prevention Study	Finland	50-69	0	6.1	21930	SFA, MUFA, PUFA	Q5 vs Q1	NA	635	NA	7
**Holmes et al., 1999[100]**	Female registered nurses	US	54	100	13	1982	total protein	Q5 vs Q1	378	NA	NA	8
**Payette et al., 1999[103]**	NA	Canada	60-94	71.9	3.5	288	total protein	NA	102	NA	NA	6
**Palli et al., 2000[105]**	GC patients	Italy	NA	37.4	11	382	total protein, animal protein, plant protein, total fat, SFA, MUFA, PUFA, animal fat, plant fat, carbohydrates	Q3 vs Q1	NA	NA	317	7
**Boniface et al., 2002[39]**	The Health and Lifestyle Survey	UK	40-75	54	16	2676	total fat, SFA, PUFA	Q5 vs Q1	NA	155	NA	6
**Hu et al., 2003[108]**	NHS	US	30-55	100	16	5130	ω-3 Fatty Acids	Q5 vs Q1	468	141	NA	8
**Sauvaget et al., 2004[41]**	AHS	Japan	57	100	14	382	animal protein, plant protein, total fat, SFA, MUFA, PUFA, animal fat, plant fat	Q3 vs Q1	NA	60	NA	7
**Borugian et al., 2004[111]**	breast cancer patients	Canada	19-25	100	10	603	total protein ,total fat	Q4 vs Q1	NA	NA	112	6
**Kelemen et al., 2005[114]**	The Iowa WHS	US	55–69	100	16.4	278	total protein,animal protein,plant protein	Q5 vs Q1	3978	739	1676	7
**Leosdottir et al.,2005[42]**	The Malmö Diet and Cancer Study	Sweden	58.3	60.6	6.6	28098	total fat, SFA, MUFA, PUFA	Q4 vs Q1	1250	339	623	9
**Solfrizzi et al.,2005[43]**	ILSA	Italy	73.0±5.5	44.6	8.5	278	total protein, total fat, SFA, MUFA, PUFA, carbohydrates	Q4 vs Q1	91	NA	NA	9
**Tucker et al., 2005[45]**	BLSA	US	62.3,34-80	0	18	501	SFA	NA	306	71	NA	8
**Trichopoulou et al,2006[117]**	Greek-EPIC Cohort	Greece	NA	48	4.5	1013	SFA,MUFA,PUFA	NA	80	46	19	10
**Lagiou et al., 2007[10]**	Scandinavian Women's Lifestyle and Health Cohort	Sweden	30–49	100	12	42237	total protein, carbohydrates	per increasing decile of protein intake	588	75	284	8

Supplemental Table S5 | Continued

author, publication year	Study cohort/ populations	Country	baseline age (years, mean or range)	female (%)	Follow-up (years)	number of participants	Exposure	Quantity (Highest vs Lowest Intake)	Number of death			NOS score
									All deaths	CVD deaths	Cancer deaths	
**Lagiou et al., 2007[10]**	Scandinavian Women's Lifestyle and Health Cohort	Sweden	30–49	100	12	42237	total protein, carbohydrates	per increasing decile of protein intake	588	75	284	8
**Xu et al.,2006[46]**	the Strong Heart Study	US	47–59	61.1	7.2±2.3	1659	total fat, SFA, MUFA, PUFA	Q4 vs Q1	NA	46	NA	8
			60–79	68.3		1279			NA	92	NA	
**Smit et al., 2007[119]**	PRHHP	US	45–64	0	12	603	total protein, animal protein, plant protein, total fat, carbohydrates	Q4 vs Q1	NA	NA	167	8
**Trichopoulou et al., 2007[11]**	EPIC	Greece	20–86	59	4.9	22944	carbohydrates	per increasing decile of protein intake	455	NA	NA	6
**Halbesma et al., 2009[121]**	NA	Netherlands	20-75	50	6.4	16922	total protein	Q5 vs Q1	443	NA	NA	9
**Bates et al., 2010[122]**	The community living population of Britain	UK	76.7	50.2	14	1100	total protein,total fat	neutral value of 0.5	749	199	NA	9
**Fung et al.,2010[9]**	NHS	US	34–59	100	26	85168	Carbohydrates	D10 vs D1	12555	2458	5780	8
	HPFS	US	40–75	0	20	44548	Carbohydrates	D10 vs D1	8678	2746	2960	
**Preis et al.,2010[123]**	HPFS	European countries	40-75	0	18	43960	total protein, animal protein, plant protein	Q5 vs Q1	NA	1155	NA	8
**Yamagishi et al.,2010[124]**	JACC	Japan	40-79	60.6	14.1	58453	SFA	Q5 vs Q1	NA	979	NA	9
**Chiuve et al.,2012[127]**	NHS	US	34-59	100	30+	91981	total fat, SFA, MUFA, PUFA	Q5 vs Q1	385	NA	NA	8
**Dilis et al.,2012[47]**	The EPIC cohort	Greek	20-86	59.3	10	23929	total fat, SFA, MUFA, PUFA	T3 vs T1	NA	240	NA	8
**Nagata et al., 2012[130]**	the Takayama Study	Japan	≥35		16	28356	total fat, SFA, MUFA, PUFA	Q5 vs Q1	4616	1429	1401	8
**Nilsson et al., 2012[8]**	Västerbotten Intervention Program	Sweden	49	51	10	77319	total protein, carbohydrates	NA	2383	681	975	8
**Argos et al., 2013[133]**	HEALS	Bangladesh	36.9	62.1	9	17244	total protein, total fat, carbohydrates	Q3 vs Q1	818	NA	135	7
**Levine et al., 2014[139]**	NHANES III	US	64.8	55.4	13.1	6381	total protein	Q3 vs Q1	2553	1212	638	8
**Miyagawa et al.,2014[140]**	NIPPON DATA	Japan	50	56.2	24	9190	n-3 PUFA	Q4 vs Q1	2551	879	NA	8
**Rebello et al.,2014[141]**	Singapore Chinese Health Study	Singapore	45-74	56	15	53469	carbohydrates	Q5 vs Q1	NA	1660	NA	8
**Wakai et al.,2014[67]**	JACC	Japan	56	60.6%	19.3	58672	total fat, SFA, MUFA, PUFA	Q5 vs Q1	11656	3393	4241	8
**Campmans-Kuijpers et al., 2015[143]**	NA	European countries	57.5	44.8	9.4	4082	total protein, animal protein, plant protein, total fat, SFA, MUFA, PUFA	Per 5% increase	787	266	NA	7
**Guasch-Ferré et al.,2015[62]**	the PREDIMED study	Spain	67±6	57.5	6	7038	total fat, SFA, MUFA, PUFA	Q5 vs Q1	516	102	NA	8
**Li et al., 2015[145]**	NHS	US	30-55	100	30	84628	total fat, SFA, MUFA, PUFA	Q5 vs Q1	NA	2736	NA	8
	HPFS	US	40-75	0	24	42908						
**Nagata et al.,2015[146]**	the Takayama Study	Japan	35-101	54	16	29079	total protein, animal protein, plant protein	Q4 vs Q1	NA	677	NA	8

Supplemental Table S5 | Continued

author, publication year	Study cohort/ populations	Country	baseline age (years, mean or range)	female (%)	Follow-up (years)	number of participants	Exposure	Quantity (Highest vs Lowest Intake)	Number of death			NOS score
									All deaths	CVD deaths	Cancer deaths	
**Puaschitz et al.,2015[147]**	WENBIT	Norway	61.7	19	4.8	2412	SFA	Q4 vs Q1	137	NA	NA	8
**Courand et al.,2016[149]**	The OLD-HTA Lyon cohort	France	45.1	42.1	10	1128	total protein	Q3 vs Q1	289	202	NA	6
**Hernández-Alonso et al., 2016[150]**	PREDIMED	Spain	55-80	57.4	4.6	7216	total protein, animal protein, plant protein	Q5 vs Q1	323	81	130	8
**Owen et al.,2016[151]**	AusDiab Study	Australia	≥25	55	12.6	11247	n-3 PUFA、n-6 PUFA	Q5 vs Q1	1265	277	NA	8
**Song et al., 2016[153]**	NHS, HPFS	US	49	64.7	27	131342	animal protein, plant protein	Q5 vs Q1	36115	8851	13159	8
**Wang et al.,2016[28]**	NHS<br>HPFS	US<br>US	30-55<br>40-75	100<br>0	30+<br>24	83349<br>42884	total fat, SFA, MUFA, PUFA	Q5 vs Q1<br>Q5 vs Q1	20314<br>12990	3960<br>3025	7018<br>4192	8
**Dehghan et al.,2017[13]**	PURE	18 countries*	50.29 ±9.92，35-70	58.3	7.4	135335	total protein, total fat, carbohydrates	Q5 vs Q1	5796	4784	NA	8
**Holmes et al.,2017[156]**	NHS	US	30-55	100	29	6348	animal protein, plant protein	Q5 vs Q1	NA	NA	919	7
**Rhee et al., 2017[157]**	WHS	US	45+	100	21	38392	α-Linolenic acid，Marine omega-3 fatty acids	Q5 vs Q1	NA	501	NA	7
**Zaslavsky et al.,2017[158]**	WHI	US	65-84	100	12.4	10034	total protein	Q4 vs Q1	3529	NA	NA	7
**Arthur et al.,2018[160]**	UM HN-SPORE	US	60.9 ±11	0.1	2.2	414	total protein, total fat, carbohydrates	high vs low	70	NA	NA	7
**Ricci et al.,2018[161]**	NHANES	US	≥30 y	50.6	6.1	18372	SFA, MUFA, PUFA	Q3 vs Q1	1118	267	289	8
**Seidelmann et al.,2018[14]**	ARIC	US	45–64	56	25	15428	carbohydrates	Q5 vs Q1	6283	NA	NA	8
**Song et al.,2018[162]**	NHS, HPFS	US	30-75	59.6	9	1542	animal protein, plant protein	animal protein per 3.4 % increase, plant protein per 1.4 % increase	817	NA	185	8
**Tharrey et al.,2018[163]**	AHS-2	US and Canada	> 25	NA	9.4	81337	animal protein,plant protein	18-g increase	NA	2276	NA	8
**Budhathoki et al.,2019[18]**	JPHC	Japan	55.7	54.5	18	70696	total protein, animal protein, plant protein	Q5 vs Q1	12381	3025	5055	8
**Chan et al., 2019[164]**	NA	China	>65	51	13.8	3020	total protein, animal protein, plant protein	Q5 vs Q1	963	205	336	7
**Jiao et al.,2019[165]**	NHS<br>HPFS	US<br>US	30-55<br>40-75	100<br>0	11<br>11	9053<br>2211	SFA,MUFA,PUFA	Q4 vs Q1<br>Q4 vs Q1	2502	646		8
**Kurihara et al.,2019[166]**	NIPPONDATA 90	Japan	52.6	58.4	13.9	7744	plant protein	Q4 vs Q1	1213	354	NA	8
**Mazidi et al.,2019[167]**	NHANES 1999–2010	US	47.6	51.4	12	24825	Low-Carbohydrate-Diet Scores	Q4 vs Q1	3432	709	827	9
**Papanikolaou et al.,2019[19]**	NHANES III	US	19-99	50	18	34398	animal protein, plant protein	NA	4280	NA	NA	8
**Virtanen et al.,2019[17]**	KIHD	Finland	52.7–53.7	0	22.31	2641	total protein, animal protein, plant protein	Q4 vs Q1	1225	618	347	7
**Zhuang et al.,2019[54]**	CHNS (1989–2011)	China	41.3	54.8	14	14383	SFA	Q4 vs Q1	1011	NA	NA	7

Supplemental Table S5 | Continued

author, publication year	Study cohort/ populations	Country	baseline age (years, mean or range)	female （%）	Follow-up (years)	number of participants	Exposure	Quantity (Highest vs Lowest Intake)	Number of death			NOS score
									All deaths	CVD deaths	Cancer deaths	
**Zhuang et al.,2019[68]**	CHNS	China	NA	NA	14	14117	PUFA	Q4 vs Q1	1007	NA	NA	8
	NHANES	US	NA	NA	14	36032	PUFA	Q4 vs Q1	4826	NA	NA	
**Zhuang et al.,2019[63]**	The NIH-AARP Diet and Health Study	US	50-71	41.2	16	521120	SFA, MUFA, PUFA	Q5 vs Q1	129328	NA	NA	7
**Chen et al.,2020[25]**	The Rotterdam Study	Netherlands	63.5	60.8	13	7786	total protein, animal protein, plant protein	Q4 vs Q1	3589	877	896	9
**Ho et al., 2020[169]**	UK Biobank	UK	56.15±7.94	55.9	10.6	195658	total protein, SFA, MUFA, PUFA	high vs low	NA	NA	NA	9
**Huang et al.,2020[170]**	The NIH-AARP Diet and Health Study	US	F:62.0(5.4); M:62.2(5.4)	43.0	16	416104	plant protein	Per 10 g/1000 kcal increment	77614	22228	10283	8
**Langsetmo et al.,2020[171]**	MrOS study	US	73.6±5.8	0.6	10	5790	protein	low vs high	1611	587	473	7
**Lelli et al.,2020[172]**	the longitudinal In CHIANTI study	Italy	75±7.3	55	9	927	PUFA/total lipid	Q4 vs Q1	NA	NA	NA	7
**Lin et al., 2020[173]**	CMUH	China	NA	NA	7.4	15289	carbohydrates	NA	2784		NA	6
**Mao et al., 2020[174]**	CHNS	China	≥20	NA	14	14305	MUFA	Q4 vs Q1	1006	NA	NA	9
**Mazidi et al.,2020[50]**	NHANES 1999-2010	US	49.6	51.5	12	24144	total fat, SFA, MUFA, PUFA	Q4 vs Q1	3632	714	NA	9
**Mendonça et al.,2020[175]**	The Newcastle 85+ Study	US	≥85	59.7	5	717	total protein	Q3 vs Q1	457	NA	NA	7
**Miyazawa et al.,2020[177]**	NIPPON DATA	Japan	30-79	56.1	24	8925	carbohydrates	Q4 vs Q1	NA	823	NA	8
**Shan et al.,2020[178]**	NHANES (1999-2014)	US	49.7 ±18.3	52.6	8	37233	Low-Carbohydrate-Diet Scores	Q5 vs Q1	4866	849	1068	8
**Trevisan et al.,2020[179]**	The Alpines Project	Italy	48.6	0	7	5049	total protein, animal protein, plant protein, total fat, SFA, MUFA, PUFA, animal fat, plant fat	Q4 vs Q1	291	NA	NA	6
**Akter et al., 2021[180]**	JPHC	Japan	NA	54.1	16.9	93654	Low-Carbohydrate-Diet Scores	Q5 vs Q1	13179	3450	5246	8
**Kwon et al.,2021[181]**	KoGES	Korea	53.8	65.3	8.15	194295	total fat	Q5 vs Q1	3866	NA	NA	7
**Sun et al., 2021[183]**	WHI	US	50-79	100	18.1	102521	animal protein, plant protein	Q5 vs Q1	25976	6993	7516	7
**Yao et al., 2021[184]**	PLCO cancer screening trial	Korea	62.4±5.3	51.4	17	101832	total fat,SFA,MUFA,PUFA	Q5 vs Q1	24141	7534	7161	7

Abbreviations:

SACCR: the South Australian Central Cancer Registry; NHANES: National Health and Nutrition Examination Survey; HPFS: The Health Professionals Follow-up Study; NHS: The Nurses' Health Study cohort. AHS: The Adult Health Study; ILSA: Italian Longitudinal Study on Aging' study; BLSA: the Baltimore Longitudinal Study of Aging; EPIC: European Prospective Investigation into Cancer and Nutrition Study; JACC: the Japan Collaborative Cohort Study; PRHHP: The Puerto Rico Heart Health Program; HEALS: the Health Effects of Arsenic Longitudinal Study; NIPPON DATA: The National Integrated Project for Prospective Observation of Non-communicable Disease And its Trends in the Aged; WENBIT: the Western Norway B-Vitamin Intervention Trial; PREDIMED : PREvención con DIeta MEDiterránea cohort; AusDiab: The Australian Diabetes, Obesity and Lifestyle Study; PURE: The Prospective Urban Rural Epidemiology study; WHS: the Women's Health Study; WHI: The Women's Health Initiative; AHS: The Adventist Health Study; JPHC: Japan Public Health Center–based Prospective Cohort Study; UM HN-SPORE: the University of Michigan Head and Neck Specialized Program of Research Excellence; NIPPONDATA90: The National Integrated Project for Prospective Observation of Non-communicable Disease and Its Trends in the Age 1990; KIHD: The Kuopio Ischaemic Heart Disease Risk Factor Study ; CHNS: the China Health and Nutrition Survey; NIH-AARP: National Institutes of Health-AARP; CMUH: China Medical University Hospital; AusDiab Study: The Australian Diabetes, Obesity and Lifestyle Study; CHNS: the China Health and Nutrition Survey; MrOS study: multicenter Osteoporotic Fractures in Men study; NIPPON DATA: The National Integrated Project for Prospective Observation of Non-communicable Disease and its Trends in the Aged ; KoGES: the Korean Genome and Epidemiology Study; PLCO cancer screening trial: the Prostate, Lung, Colorectal, and Ovarian cancer screening trial. *from 18 countries, three high-income (Canada, Sweden, and United Arab Emirates), 11 middle-income (Argentina, Brazil, Chile, China, Colombia, Iran, Malaysia, occupied Palestinian territory, Poland, South Africa, and Turkey) and four low-income countries (Bangladesh, India, Pakistan, and Zimbabwe).

Table S6. Variety of Macronutrients intake assessment and outcome methods

author，publication year	Diet assessment method	Outcome assessment method
**Protein Intake with All-Cause and Cause-Specific Mortality**		
Rohan et al.,1993	Self-administered FFQ	Determine the patient's life status by linking the research files with the files saved by SACCR through computer records
Dwyer et al.,1994	24-Hour Food Consumption Intake	The records of participants' mortality were obtained by linkage to the National Death
Esrey et al.,1996	24-Hour Food Consumption Intake	The records of participants' mortality were obtained by CDC and NCHS
Payette et al.,1999	3-day weighted food records of 3 consecutive days of the week	CLSC in combination with information from medical, hospital,or nursing home records and surveillance of obituaries. Official death certificates (Ministere de la Sante et des Services Sociaux[MSSS], Quebec) were obtained for confirmation of death.
Holmes et al.,1999	Validated, semi-quantitative 126-item FFQ	Medical record, autopsy report, death certificate, US postal system, state vital statistics departments, National Death Index.
Palli et al.,2000	Self-administered FFQ	Vital status information was sought for all patients by periodic linkage to Municipal Population Offices and to the Regional Mortality Registry on December 31,1997.
Sauvaget et al.,2004	24-Hour Food Consumption Intake	Vital status was ascertained by linkage to the nationwide family registration system of Japan.
Borugian et al.,2004	Self-administered FFQ	outcomes were ascertained by linkage to the BC Cancer Registry after 10 years of follow-up.
Kelemen et al.,2005	Self-administered FFQ	Death certificates. Residencial registry to confirm residence status
Solfrizzi et al.,2005	Validated, semi-quantitative 77-item FFQ	Death certificates. Residencial registry to confirm residence status
Smit et al.,2007	24-Hour Food Consumption Intake	participants in the PRHHP with the Puerto Rico Cancer Registry and Puerto Rico Vital Statistics Registry.
Lagiou et al.,2007	Self-administered FFQ	Linkages with the Swedish nationwide health registers, by means of the unique per individual Swedish national registration number, were used for the follow up of the cohort with respect to death and emigration.
Halbesma et al.,2009	Self-administered FFQ	Data on mortality were received through the municipal register. Cause of death was obtained by linking the number of the death certificate to the primary cause of death as coded by the Dutch Central Bureau of Statistics.
Bates et al.,2010	24-Hour Food Consumption Intake	Death certificates. Residencial registry to confirm residence status
Preis et al.,2010	Validated, semi- quantitative 126- item FFQ	Medical record, autopsy report, death certificate, US postal system, state vital statistics departments, National Death Index.
Nilsson et al.,2012	Self-administered FFQ	Mortality end-points were identified by linking the VIP database with the Swedish national cause-of-death registry
Argos et al.,2013	Self-administered FFQ	Death certificates. Residencial registry to confirm residence status

Levine et al.,2014	24-h dietary recalls by trained interviewers	Dietary data and mortality data were linked using different institutional database: WWEIA; NCHS; NDI
Nagata et al.,2015	Self-administered FFQ	Death certificates. Residencial registry to confirm residence status
Campmans-Kuijpers et al.,2015	Self-administered FFQ	Information on vital status, cause and date of death, were obtained by using follow-up mailings and subsequent inquiries to municipal registries, regional health departments, physicians, or by record linkages with local, regional, or central cancer registries, boards of health, or hospitals (Germany), or death indexes (other countries).
Courand et al.,2016	Self-administered FFQ	Deaths at 10 years of follow-up were obtained from the Répertoire National d'Identification des Personnes Physiques (a directory maintained by the Institut National de la Statistique et des Etudes Economiques).
Hernández-Alonso et al.,2016	Validated, semi-quantitative 137-item FFQ	Death certificates. Residencial registry to confirm residence status
Song et al.,2016	Validated, semi- quantitative 126- item FFQ	Medical record, autopsy report, death certificate, US postal system, state vital statistics departments, National Death Index.
Holmes et al.,2017	Validated, semi- quantitative 126- item FFQ	Medical record, autopsy report, death certificate, US postal system, state vital statistics departments, National Death Index.
Zaslavsky et al.,2017	Self-administered FFQ	Participants' deaths were adjudicated by study physicians with the use of hospital records, autopsy or coroner reports, and/or death certificates
Dehghan et al.,2017	Self-administered FFQ	Death certificates. Residencial registry to confirm residence status
Arthur et al.,2018	Self-administered FFQ	Deaths were captured through the Social Security Death Index, yearly survey updates, notification from family or medical record reviews
Tharrey et al.,2018	Self-administered FFQ	biennial follow-up of participants and linkage with the National Death Index
Song et al.,2018	Validated, semi- quantitative 126- item FFQ	Medical record, autopsy report, death certificate, US postal system, state vital statistics departments, National Death Index.
Mendonça et al.,2020	24-h dietary recalls by trained interviewers	Information on date of death was obtained from NHS Digital, UK.
Kurihara et al.,2019	24-h dietary recalls by trained interviewers	Medical record, autopsy report, death certificate, US postal system, state vital statistics departments, National Death Index.
Virtanen et al.,2019	4-d dietary records	Deaths were ascertained by a computer linkage to the national Causes of Death Register with the use of the Finnish personal identification code (social security number).
Budhathoki et al.,2019	Self-administered FFQ	Residential and vital statuses of cohort participants during follow-up were determined annually through the residential registry.
Chan et al.,2019	Self-administered FFQ	Mortality data were ascertained from the Hong Kong Government Death Registry.
Ho et al.,2020	24-h dietary recalls by trained interviewers	Date of death was obtained from death certificates held within the NHS Information Centre (England and Wales) and the NHS Central Register Scotland (Scotland).
Langsetmo et al.,2020	Self-administered FFQ	Deaths were reported by proxy respondent and were confirmed with hospital discharge records and/or death certificate.

Chen et al.,2020	**a semi-quantitative food questionnaire (FFQ)**	Information on vital status of the participants was obtained from clinical follow-up data collection and from munici pal records.
Huang et al.,2020	Diet History Questionnaire	Vital status of participants was ascertained via linkage with the Social Security Administration Death Master File, and specific causes of death were identified through linkage with the National Death Index Plus, updated by the National Center for Health Statistics
Trevisan et al.,2020	Self-administered FFQ	Deaths were reported by proxy respondent and were confirmed with hospital discharge records and/or death certificate.
Sun et al.,2021	Self-administered FFQ	Deaths were ascertained by reviewing death certificates, medical records, autopsy reports, or by linkage to the National Death Index
	**Protein Intake with CVD Morbidity**	
Hu et al.,1999	Validated, semi-quantitative 126- item FFQ	Self-reported
Zhang et al.,2003	Self-administered FFQ	Self-reported
Iso et al.,2001	Validated, semi- quantitative 126- item FFQ	Hospital Discharge Register
Larsson et al.,2012	Validated, semi-quantitative 96- item FFQ	ascertained by linkage of the study cohort to the Swedish Hospital Discharge Registry, which provides virtually complete national coverage
Preis et al.,2010	Validated, semi-quantitative 131- item FFQ	participant reported a diagnosis and hospitalization for MI
Wallstrom et al.,2012	an interview-based, modified diet history method that combined	Self-reported
Haring et al.,2014	Validated, semi-quantitative 66- item FFQ	CHD events were identified and adjudicated using information from study visits, yearly telephone follow-up calls, review of hospital discharge lists and medical charts, death certificates, next-of-kin interviews, and physician-completed questionnaires
Xu et al.,2016	7-day dietary record	The Swedish National vital status registry and Swedish National Hospital discharge register were used to identify CVD events (identified by ICD-9 codes 390–459 or ICD-10 codes I00–I99), and were thereafter validated by inspection of medical records.
Dehghan et al.,2017	Self-administered FFQ	Death certificates. Residencial registry to confirm residence status
Okada et al.,2019	24-h dietary recalls by trained interviewers	Death certificates, national insurance claims, reports from local physicians, reports from public health nurses and health volunteers, and an annual cardiovascular risk survey were used to ascertain the endpoint of stroke.
	**Fat Intake with All-Cause and Cause-Specific Mortality**	
Posner et al.,1991	24-Hour Food Consumption Intake	Medical record, autopsy report, death certificate, US postal system, state vital statistics departments, National Death Index.
Rohan et al.,1993	Self-administered FFQ	Determine the patient's life status by linking the research files with the files saved by SACCR through computer records

Esrey et al.,1996	24-Hour Food Consumption Intake	The records of participants' mortality were obtained by CDC and NCHS
Mann et al.,1997	Self-administered FFQ	Flagging of each subject's medical records at the National Health Service Central Register enabled death certificates to be sent to the investigators following the death of participants.
Pietinen et al.,1997	Self-administered FFQ	Data on nonfatal myocardial infarction were obtained from the National Hospital Discharge Register.
Boniface et al.,2002	Self-administered FFQ	Death certificates. Residencial registry to confirm residence status
Palli et al.,2000	Self-administered FFQ	Vital status information was sought for all patients by periodic linkage to Municipal Population Offices and to the Regional Mortality Registry on December 31,1997.
Hu et al.,2003	Validated, semi-quantitative 126- item FFQ	Medical record, autopsy report, death certificate, US postal system, state vital statistics departments, National Death Index.
Borugian et al.,2004	Self-administered FFQ	outcomes were ascertained by linkage to the BC Cancer Registry after 10 years of follow-up.
Sauvaget et al.,2004	24-Hour Food Consumption Intake	Vital status was ascertained by linkage to the nationwide family registration system of Japan.
Solfrizzi et al.,2005	Validated, semi-quantitative 77-item FFQ	Death certificates. Residencial registry to confirm residence status
Leosdottir et al.,2005	Self-administered FFQ	Information on prior medical history was acquired from the questionnaire (for diabetes) and from local or national registries (for diagnosis of cancer, myocardial infarction, or stroke).
Tucker et al.,2005	7-d diet record	Death certificates. Residencial registry to confirm residence status
Xu et al.,2006	24-h dietary recall	CHD events that occurred during the follow-up period were ascertained from the annual mortality and morbidity surveillance or at the third examination (1998–1999)
Trichopoulou et al. 2007	Self-administered FFQ	Death certificates. Residencial registry to confirm residence status
Smit et al.,2007	24-Hour Food Consumption Intake	Death certificates. Residencial registry to confirm residence status
Bates et al.,2010	24-Hour Food Consumption Intake	Death certificates. Residencial registry to confirm residence status
Yamagishi et al.,2010	Self-administered FFQ	all deaths that occurred in the cohort were ascertained by death certificates from a public health center
Dilis et al.,2012	a validated interviewer-administered semi-quantitative FFQ	Self-reporting of a CHD event during the follow-up was confirmed through hospital discharge data, medical records or death certificates
Nagata et al.,2012	a validated interviewer-administered semi-quantitative FFQ	confirmed with data from the National Vital Statistics provided by the Ministry of Health, Labor, and Welfare.
Chiuve et al.,2012	Validated, semi- quantitative 126- item FFQ	Medical record, autopsy report, death certificate, US postal system, state vital statistics departments, National Death Index.
Argos et al.,2013	Self-administered FFQ	Death certificates. Residencial registry to confirm residence status
Wakai et al.,2014	Self-administered FFQ	We used population registries from the involved municipalities to determine the vital and residential status of participants.
Miyagawa et al.,2014	Self-administered FFQ	Death certificates.<br>Residencial registry to confirm residence status
Puaschitz et al.,2015	a semiquantitative FFQ	Clinical data were obtained from hospitals and information on deaths from the Norwegian Cause of Death Registry

Owen et al.,2016	Validated, semi- quantitative 126- item FFQ	Death certificates. Residencial registry to confirm residence status
Guasch-Ferré et al.,2015	Self-administered FFQ	Death certificates. Residencial registry to confirm residence status
Campmans-Kuijpers et al.,2015	Self-administered FFQ	Information on vital status, cause and date of death, were obtained by using follow-up mailings and subsequent inquiries to municipal registries, regional health departments, physicians, or by record linkages with local, regional, or central cancer registries, boards of health, or hospitals (Germany), or death indexes (other countries).
Li et al.,2015	Validated, semi- quantitative 126- item FFQ	Medical record, autopsy report, death certificate, US postal system, state vital statistics departments, National Death Index.
Wang et al.,2016	Validated, semi- quantitative 126- item FFQ	Medical record, autopsy report, death certificate, US postal system, state vital statistics departments, National Death Index.
Rhee et al.,2017	Self-administered FFQ	self-reported data on incident physician diagnoses of cardiovascular events; autopsy reports, death certificates, medical records, or information obtained from next of kin or family members
Dehghan et al.,2017	Self-administered FFQ	Death certificates. Residencial registry to confirm residence status
Ricci et al.,2018	24-h dietary recalls by trained interviewers	Dietary data and mortality data were linked using different institutional database: WWEIA; NCHS; NDI
Arthur et al.,2018	Self-administered FFQ	Deaths were captured through the Social Security Death Index, yearly survey updates, notification from family or medical record reviews
Jiao et al.,2019	Validated, semi- quantitative 126- item FFQ	Medical record, autopsy report, death certificate, US postal system, state vital statistics departments, National Death Index.
Zhuang et al.,2019 b	3 consecutive 24-h dietary recalls	Death status was ascertained by the report of household members in each survey.
Zhuang et al.,2019 a	Self-administered FFQ	Medical record, autopsy report, death certificate, US postal system, state vital statistics departments, National Death Index.
Zhuang et al.,2019 c	24-h dietary recalls by trained interviewers	Dietary data and mortality data were linked using different institutional database: WWEIA; NCHS; NDI
Mao et al.,2020	3 consecutive 24-h dietary recalls	The death date in every round of survey was recorded based on the report of household members in each survey and denoted in CHNS database.
Lelli et al.,2020	Self-administered FFQ	mortality registers after a 9-year follow-up
Ho et al.,2020	24-h dietary recalls by trained interviewers	Date of death was obtained from death certificates held within the NHS Information Centre (England and Wales) and the NHS Central Register Scotland (Scotland).
Mazidi et al.,2020	24-h dietary recalls by trained interviewers	Dietary data and mortality data were linked using different institutional database: WWEIA; NCHS; NDI
Trevisan et al.,2020	Self-administered FFQ	Deaths were reported by proxy respondent and were confirmed with hospital discharge records and/or death certificate.
Kwon et al.,2021	Validated, semi- quantitative 123- item FFQ	Mortality status and causes of death were determined using publicly accessible files in the KoGES-linked National Death Index as a reference
Yao et al.,2021	Self-administered FFQ	Deaths were identified by annual mailed questionnaires and periodic linkage to the National Death Index.

**Fat Intake with CVD Morbidity**		
McGee et al.,1984	24-h dietary recalls by trained interviewers	Self-reported
Fehily et al.,1993	Self-administered FFQ	Self-reported
Goldbourt et al.,1993	Self-administered FFQ	Self-reported
Ascherio et al.,1995	Validated, semi- quantitative 126- item FFQ	Self-reported
Ascherio et al.,1996	Validated, semi- quantitative 126- item FFQ	Self-reported
Gillman et al.,1997		
Hu et al.,1997	Validated, semi- quantitative 126- item FFQ	Self-reported
Seino et al.,1997	Self-administered FFQ	Self-reported
Pietinen et al.,1997	Self-administered FFQ	Hospital Discharge Register
Hu et al.,1999	Validated, semi- quantitative 126- item FFQ	Self-reported
Iso et al.,2001	Validated, semi- quantitative 126- item FFQ	Hospital Discharge Register
He et al.,2003	Self-administered FFQ	postal authorities or the national death index
Hu et al.,2003	Validated, semi- quantitative 126- item FFQ	Medical record, autopsy report, death certificate, US postal system, state vital statistics departments, National Death Index.
Iso et al.,2003	24-h dietary recalls by trained interviewers	1) national insurance claims, 2) reports by local physicians, 3) ambulance records, 4) death certificates, 5) reports by public health nurses and health volunteers, and 6) cardiovascular risk surveys.
Jakobsen et al.,2004	a 7-day weighed food record	record linkage to the National Patient Registry
Tanasescu et al.,2004	Validated, semi- quantitative 126- item FFQ	Self-reported
Oh et al.,2005	Validated, semi- quantitative 126- item FFQ	Self-reported
Trichopoulou et al. 2007	Self-administered FFQ	Death certificates. Residencial registry to confirm residence status
Xu et al.,2006	24-h dietary recall	CHD events that occurred during the follow-up period were ascertained from the annual mortality and morbidity surveillance or at the third examination (1998–1999)
Leosdottir et al.,2007	a 7-day weighed food record	Self-reported

Boden-Albala et al.,2009	Self-administered FFQ	Hospital Discharge Register
Yamagishi et al.,2010	Self-administered FFQ	all deaths that occurred in the cohort were ascertained by death certificates from a public health center
Houston et al.,2011	Self-administered FFQ	Hospital Discharge Register
Atkinson et al.,2011	Self-administered FFQ	Self-reported
Dilis et al.,2012	a validated interviewer-administered semi-quantitative FFQ	Self-reporting of a CHD event during the follow-up was confirmed through hospital discharge data, medical records or death certificates
Wallstrom et al.,2012	an interview-based, modified diet history method that combined	Self-reported
Larsson et al.,2012	Validated, semi- quantitative 96- item FFQ	ascertained by linkage of the study cohort to the Swedish Hospital Discharge Registry, which provides virtually complete national coverage
Yaemsiri et al.,2012	Self-administered FFQ	Self-reported
de Oliveira et al.,2012	Self-administered FFQ	Self-reported
Yamagishi et al.,2013	Self-administered FFQ	Hospital Discharge Register
Virtanen et al.,2014	24-h dietary recall	Hospital Discharge Register
Santos et al.,2014	a 3-day WDR technique (two nonconsecutive weekdays and one day of the weekend)	Self-reported
Chiuve et al.,2015	Self-administered FFQ	Self-reported
Puaschitz et al.,2015	Self-administered FFQ	Clinical data were obtained from hospitals and information on deaths from the Norwegian Cause of Death Registry
Li et al.,2015	Validated, semi- quantitative 126- item FFQ	Self-reported
Guasch-Ferré et al.,2015	Self-administered FFQ	Death certificates. Residencial registry to confirm residence status
Chen et al.,2016	Validated, semi- quantitative 126- item FFQ	Hospital Discharge Register
Rhee et al.,2017	Self-administered FFQ	self-reported data on incident physician diagnoses of cardiovascular events; autopsy reports, death certificates, medical records, or information obtained from next of kin or family members
Praagman et al.,2016	Self-administered FFQ	the Dutch Center for Health Care Information
Zong et al.,2016	Validated, semi- quantitative 126- item FFQ	Self-reported
Dehghan et al.,2017	Self-administered FFQ	Death certificates. Residencial registry to confirm residence status

Okada et al.,2019	24-h dietary recalls by trained interviewers	Death certificates, national insurance claims, reports from local physicians, reports from public health nurses and health volunteers, and an annual cardiovascular risk survey were used to ascertain the endpoint of stroke.
Mirmiran et al.,2020	Self-administered FFQ	telephone call follow-ups
Sadeghi et al.,2021	Self-administered FFQ	medical records, hospital records, death certificates, and verbal autopsies
**carbohydrate Intake with All-Cause and Cause-Specific Mortality**		
Rohan et al.,1993	Self-administered FFQ	Determine the patient's life status by linking the research files with the files saved by SACCR through computer records
Esrey et al.,1996	24-Hour Food Consumption Intake	The records of participants' mortality were obtained by CDC and NCHS
Palli et al.,2000	Self-administered FFQ	Vital status information was sought for all patients by periodic linkage to Municipal Population Offices and to the Regional Mortality Registry on December 31,1997.
Solfrizzi et al.,2005	Validated, semi-quantitative 77-item FFQ	Death certificates. Residencial registry to confirm residence status
Lagiou et al.,2007	Self-administered FFQ	Linkages with the Swedish nationwide health registers, by means of the unique per individual Swedish national registration number, were used for the follow up of the cohort with respect to death and emigration.
Trichopoulou et al. 2007	Self-administered FFQ	Death certificates. Residencial registry to confirm residence status
Smit et al.,2007	24-Hour Food Consumption Intake	Death certificates. Residencial registry to confirm residence status
Fung et al.,2010	Validated, semi- quantitative 126- item FFQ	Medical record, autopsy report, death certificate, US postal system, state vital statistics departments, National Death Index.
Nilsson et al.,2012	Self-administered FFQ	Mortality end-points were identified by linking the VIP database with the Swedish national cause-of-death registry
Rebello et al.,2014	Validated, semi- quantitative 165- item FFQ	The cohort is followed up for mortality through regular record linkage with the population-based Singapore Registry of Births and Deaths.
Dehghan et al.,2017	Self-administered FFQ	Death certificates. Residencial registry to confirm residence status
Seidelmann et al.,2018	Validated, semi- quantitative 66- item FFQ	Number of deaths was determined with annual (or later, semi-annual) telephone calls, linkage to local hospital and state health department records, or for those lost to follow-up, linkage to the National Death Index.
Arthur et al.,2018	Self-administered FFQ	Deaths were captured through the Social Security Death Index, yearly survey updates, notification from family or medical record reviews
Shan et al.,2020	24-h dietary recalls by trained interviewers	Dietary data and mortality data were linked using different institutional database: WWEIA; NCHS; NDI
Miyazawa et al.,2020	Self-administered FFQ	Death certificates. Residencial registry to confirm residence status
Mazidi et al.,2020	24-h dietary recalls by trained interviewers	Dietary data and mortality data were linked using different institutional database: WWEIA; NCHS; NDI
Lin et al.,2020	24-h dietary recalls by trained interviewers	annual record linkage with the National Death Datasets, by using personal identification number and date of birth provided by the Taiwan Ministry of Health and Welfare
Akter et al.,2021	Validated, semi-quantitative 147- item FFQ	We followed up the participants' residency and vital status from the second survey to 31 December 2014 using the residential registry.

		**carbohydrate Intake with CVD Morbidity**
Liu et al.,2000	Validated, semi- quantitative 126- item FFQ	Self-reported
Oh et al.,2005	Validated, semi- quantitative 126- item FFQ	Self-reported
Wallstrom et al.,2012	an interview-based, modified diet history method that combined	Self-reported
Simila et al.,2013	Self-administered FFQ	identified from the National Hospital Discharge Register and the National Register of Causes of Death
Yu et al.,2013	Self-administered FFQ	Self-reported
Li et al.,2015	Validated, semi- quantitative 126- item FFQ	Medical record, autopsy report, death certificate, US postal system, state vital statistics departments, National Death Index.
Dehghan et al.,2017	Self-administered FFQ	Death certificates. Residencial registry to confirm residence status
AlEssa et al.,2018	Validated, semi- quantitative 126- item FFQ	Self-reported
Okada et al.,2019	24-h dietary recalls by trained interviewers	Death certificates, national insurance claims, reports from local physicians, reports from public health nurses and health volunteers, and an annual cardiovascular risk survey were used to ascertain the endpoint of stroke.

Table S7. Confounding variables of included prospective cohort studies.

Study	cohort	Dietary Intake	primary confounding variable	Basic factors				lifestyle factors			socioeconomic status		Disease History				
			Energy intake	Age	Sex	Race/ethnicity	BMI / Body Weight / WC	Physical activity	Smoking	Alcohol	Education	Household income Income-to-poverty ratio	Diabetes/ glucose intolerance	cancer	CVD	hypertension/blood pressure	Medications use
McGee et al.,1984	The Honolulu Heart Program		√	√	√		√	√	√							√	
Posner et al.,1991	The Framingham Study		√	√	√			√	√	√			√			√	
Rohan et al.,1993	SACCR		√	√	√												
Fehily et al.,1993	the Caerphilly Study			√	√		√		√								
Goldbourt et al.,1993	NA		√	√	√		√	√	√							√	
Dwyer et al.,1994	NHANES I			√	√	√	√				√		√				
Ascherio et al.,1995	HPFS	√	√	√	√		√	√	√	√			√			√	
Ascherio et al.,1996	HPFS	√	√	√	√		√	√	√	√			√			√	
Esrey et al.,1996	NA		√	√	√		√		√				√			√	
Gillman et al.,1997	United States Framingham Heart Study		√	√	√												
Hu et al.,1997	NHS	√	√	√	√		√	√	√	√						√	
Seino et al.,1997	The Shibata Study			√	√											√	
Pietinen et al.,1997	The Alpha-Tocopherol, Beta-Carotene Cancer Prevention Study	√	√	√	√		√	√	√	√	√					√	
Mann et al.,1997	National Health Service Central Register			√	√				√		√	√					
Payette et al.,1999	NA			√	√		√										

Holmes et al.,1999	NHS		√	√	√				√					√			√
Hu et al.,1999	NHS	√	√	√	√		√	√	√	√					√		√
Hu et al.,1999	NHS	√	√	√	√		√	√	√	√					√		√
Palli et al.,2000	GC patients			√	√						√	√		√			
Liu et al.,2000	NHS	√	√	√	√		√	√	√	√						√	√
Iso et al.,2001	NHS	√	√	√	√		√	√	√	√	√	√	√			√	√
Boniface et al.,2002	The Health and Lifestyle Survey			√	√												
Hu et al.,2003	NHS	√	√	√	√		√	√	√	√			√	√	√	√	√
Zhang et al.,2003	SWHS	√	√	√	√		√	√	√	√	√	√				√	
He et al.,2003	HPFS	√	√	√	√		√	√	√	√						√	
Iso et al.,2003	NHS	√	√	√	√		√	√	√	√			√			√	
Sauvaget et al.,2004	AHS			√	√	√	√		√	√			√			√	
Borugian et al.,2004	breast cancer patients			√	√		√	√	√	√	√	√	√	√	√	√	
Jakobsen et al.,2004	the Research Centre for Prevention and Health	√	√	√	√		√	√	√	√			√			√	
Tanasescu et al.,2004	NHS	√	√	√	√		√	√	√	√							
Kelemen et al.,2005	IWHS	√		√	√		√	√	√	√	√			√		√	√
Solfrizzi et al.,2005	ILSA	√	√	√	√		√	√	√	√							
Leosdottir et al.,2005	The Malmö Diet and Cancer Study	√	√	√	√		√	√	√	√	√	√				√	
Tucker et al.,2005	BLSA	√		√	√		√	√	√	√							
Oh et al.,2005	NHS	√	√	√	√		√	√	√	√			√			√	√
Xu et al.,2006	the Strong Heart Study	√	√	√	√				√	√			√			√	
Trichopoulou et al. 2006																	
Smit et al.,2007	PRHHP		√	√	√		√	√	√		√						

Lagiou et al.,2007	Scandinavian Women's Lifestyle and Health Cohort	√	√	√	√	√	√	√	√	√						
Trichopoulou et al. 2007	Greek-EPIC Cohort	√	√	√	√	√	√	√	√	√					√	
Leosdottir et al.,2007	The Malmö Diet and Cancer Study	√		√	√	√	√	√	√	√	√				√	
Halbesma et al.,2009	NA			√	√	√	√	√	√							
Boden-Albala et al.,2009	the Northern Manhattan Study			√	√	√	√	√	√	√						
Bates et al.,2010	The community-living population of mainland Britain		√	√	√	√	√	√	√		√					
Preis et al.,2010	HFSP		√	√	√	√	√	√	√			√			√	
Yamagishi et al.,2010	The Japan Collaborative Cohort Study	√	√	√	√	√	√	√	√	√		√			√	
Fung et al.,2010	NHS、HPFS		√	√	√	√	√	√	√		√				√	√
Houston et al.,2011	the Health ABC Study	√	√	√	√	√	√	√	√	√		√			√	√
Atkinson et al.,2011	the Caerphilly cohort	√		√	√	√	√	√	√	√	√	√	√		√	
Nilsson et al.,2012	Västerbotten Intervention Programme	√	√	√	√	√	√	√	√							
Dilis et al.,2012	The EPIC cohort		√	√	√	√	√	√	√	√					√	
Nagata et al.,2012	the Takayama Study	√	√	√	√	√	√	√	√	√		√			√	
Chiuve et al.,2012	NHS		√	√	√	√	√	√	√			√	√	√	√	√
Larsson et al.,2012	Swedish Mammography Cohort	√	√	√	√	√	√	√	√	√		√			√	√
Wallstrom et al.,2012	Swedish population-based Malmö Diet and Cancer cohort	√	√	√	√	√	√	√	√	√						

Larsson et al.,2012	Swedish Mammography Cohort	√	√	√	√		√	√	√	√	√		√	√	√
Yaemsiri et al.,2012	the WHI-OS	√	√	√	√	√	√	√	√	√	√		√	√	√
de Oliveira et al.,2012	United States MESA Cohort	√	√	√	√	√	√	√	√	√	√				√
Argos et al.,2013	HEALS			√	√		√	√	√	√	√				
Yamagishi et al.,2013	The Japan Public Health Center-based prospective Study	√	√	√	√		√	√	√	√					
Simila et al.,2013	ATBC Study	√	√	√	√		√	√	√	√				√	
Yu et al.,2013	China Shanghai study	√	√	√	√		√	√	√	√	√	√		√	
Levine et al.,2014	NHANES III		√	√	√	√	√	√	√	√					
Wakai et al.,2014	The Japan Collaborative Cohort Study	√	√	√	√		√	√	√	√	√				
Miyagawa et al.,2014	NIPPON DATA	√	√	√	√		√	√	√	√	√	√		√	
Rebello et al.,2014	Singapore Chinese Health Study	√	√	√	√		√	√	√	√	√		√	√	√
Haring et al.,2014	ARIC	√	√	√	√		√	√	√	√	√	√		√	
Virtanen et al.,2014	KIHD	√	√	√	√		√	√	√	√	√		√	√	
Santos et al.,2014	the Diabetes research outpatient clinic at Hospital de Clínicas de Porto Alegre	√		√	√				√				√		
Nagata et al.,2015	the Takayama Study	√	√	√	√		√	√	√	√	√		√	√	√
Campmans-Kuijpers et al.,2015	EPIC	√	√	√	√		√	√	√	√	√		√		
Puaschitz et al.,2015	WENBIT	√		√	√				√				√	√	√

Chiuve et al.,2015	WHS		√	√	√		√	√	√	√	√		√			√	
Li et al.,2015	NHS HPFS	√	√	√	√		√	√	√	√			√			√	
Guasch-Ferré et al.,2015	the PREDIMED study	√	√	√	√		√	√	√	√	√	√	√			√	√
Courand et al.,2016	The OLD-HTA Lyon cohort			√	√		√		√				√			√	
Hernández-Alonso et al.,2016	PREDIMED		√	√	√		√	√	√	√			√			√	√
Song et al.,2016	NHS / HPFS	√	√	√	√		√	√	√	√						√	
Owen et al.,2016	The Australian Diabetes, Obesity and Lifestyle Study (AusDiab)		√	√	√		√	√	√	√	√		√		√		
Wang et al.,2016	NHS HPFS	√	√	√	√	√	√	√	√	√			√	√		√	
Xu et al.,2016	ULSAM	√	√	√	√		√	√	√	√			√			√	
Chen et al.,2016	the HPFS、NHS、NHSII	√	√	√	√		√						√			√	√
Praagman et al.,2016	the European Prospective Investigation into Cancer and Nutrition-Netherlands cohort		√	√	√		√	√	√	√	√						
Zong et al.,2016	the HPFS、NHS	√	√	√	√		√	√	√	√						√	√
Holmes et al.,2017	NHS			√	√		√			√							√
Zaslavsky et al.,2017	WHI	√	√	√	√	√	√	√	√	√	√	√					
Dehghan et al.,2017	PURE		√	√	√		√	√	√	√			√				
Rhee et al.,2017	WHS	√	√	√	√		√	√	√	√			√			√	√
Arthur et al.,2018	UM HN-SPORE	√	√	√	√		√	√	√					√			
Tharrey et al.,2018	AHS-2		√	√	√	√	√	√	√	√	√	√					
Song et al.,2018	NHS / HPFS	√	√	√	√		√	√	√	√							√
Ricci et al.,2018	NHANES	√		√	√	√	√	√	√	√	√					√	

Seidelmann et al.,2018	ARIC	√	√	√	√		√	√	√	√	√	√	√				
AlEssa et al.,2018	NHS、HPFS	√	√	√	√		√	√	√	√							√
Mazidi et al.,2019	NHANES 1999-2010																
Papanikolaou et al.,2019	NHANES III																
Kurihara et al.,2019	NIPPONDATA90	√	√	√	√		√	√	√	√							
Virtanen et al.,2019	KIHD	√	√	√	√		√	√	√	√	√	√	√	√	√	√	
Budhathoki et al.,2019	JPHC	√	√	√	√		√	√	√	√	√	√					
Chan et al.,2019	NA	√	√	√	√		√	√	√	√	√		√	√	√	√	
Jiao et al.,2019	NHS HPFS	√	√	√	√		√	√	√	√	√		√	√		√	
Zhuang et al.,2019 b	CHNS	√	√	√	√		√	√	√	√	√	√					
Zhuang et al.,2019 a	the National Institutes of Health-AARP Diet and Health Study	√		√	√	√	√	√	√	√	√	√	√	√		√	√
Zhuang et al.,2019 c	CHNS、NHANES	√	√	√	√		√	√	√	√	√	√					
Okada et al.,2019	CIRCS	√	√	√	√		√	√	√	√	√		√			√	
Mendonça et al.,2020	The Newcastle 85+ Study		√	√	√			√			√		√	√	√	√	
Ho et al.,2020	UK Biobank		√	√	√		√	√	√	√	√	√	√				
Langsetmo et al.,2020	MrOS		√	√	√	√	√	√	√	√	√		√	√	√	√	
Chen et al.,2020	The Rotterdam Study	√	√	√	√		√	√	√	√	√	√					
Huang et al.,2020	NIH-AARP Diet and Health Study	√	√	√	√	√	√	√	√	√	√	√	√				
Trevisan et al.,2020	The Alpines Project		√	√	√				√		√					√	
Mao et al.,2020	CHNS	√	√	√	√		√	√	√	√	√	√	√			√	
Lelli et al.,2020	the longitudinal InCHIANTI study		√	√	√		√	√	√	√			√			√	
Ho et al.,2020	UK Biobank		√	√	√		√	√	√	√	√	√	√				

Mazidi et al.,2020	NHANES 1999-2010	√	√	√	√		√	√	√	√	√		√			√	
Shan et al.,2020	NHANES(1999-2014)		√	√	√	√	√	√	√	√	√	√	√		√	√	
Miyazawa et al.,2020	NIPPON DATA	√	√	√	√		√	√	√	√			√			√	
Lin et al.,2020	CMUH		√	√	√		√	√	√	√	√	√	√				
Mirmiran et al.,2020	the Tehran Lipid and Glucose Prospective Study	√	√	√	√		√	√	√	√			√			√	√
Kwon et al.,2021	KoGES		√	√	√		√	√	√	√	√		√			√	
Yao et al.,2021	PLCO	√	√	√	√	√	√	√	√	√	√		√			√	√
Akter et al.,2021	JPHC study	√	√	√	√		√	√	√	√		√	√			√	
Sadeghi et al.,2021	ICS	√	√	√	√	√	√	√	√	√			√			√	√
Sun et al.,2021	WHI	√	√	√	√		√	√	√	√	√		√	√	√	√	

Abbreviations: BPRHS = Boston Puerto Rican Health Study, BMI = body mass index, FICSIT = Frailty and Injuries: Cooperative Studies of Intervention Techniques, NHANES = National Health and Nutrition Examination Survey, TLGS = Tehran Lipid and Glucose Study, VF = vegetable/fruit, WC = waist circumference.

Abbreviations: ADDITION = Anglo-Danish-Dutch Study of Intensive Treatment In People with Screen-Detected Diabetes in Primary Care-Cambridge study, BMI = body mass index, HPFS = Health Professionals Follow-up Study, JPHC = Japan public health center study, MORGEN = The Monitoring Project on Risk Factors and Chronic Diseases in the Netherlands Study, NHANES = National Health and Nutrition Examination Survey, NHS = Nurses Health Study, PREDIMED-Plus = PREvención con DIeta MEDiterránea (Prevention with Mediterranean Diet)-Plus, VF = vegetable/fruit

Table S8. Newcastle-Ottawa Scale (NOS) scores of included prospective cohort studies

**Reference (Last name et al., Year)**	**Selection (max 4)[a]**				**Outcome (max 3)[b]**			**Comparability (max 2)[c]**		**Total[d]**
	Representativeness of the exposed cohort	Selection of the non-exposed cohort	Ascertainment of exposure	Demonstration that outcome of interest was not present at start of study	Assessment of outcome	Was follow-up long enough for outcomes to occur	Adequacy of follow-up of cohort	Study controls for energy	Study controls for pre-specified secondary covariates	
McGee et al.,1984	1	1	1	1	1	1	0	1	1	8
Posner et al.,1991	1	1	1	1	1	1	0	1	1	8
Rohan et al.,1993	0	1	1	1	1	1	0	1	0	7
Fehily et al.,1993	1	1	1	1	1	1	0	0	1	7
Goldbourt et al.,1993	0	1	1	1	1	1	0	0	1	6
Dwyer et al.,1994	1	1	1	1	1	1	1	1	1	9
Ascherio et al.,1995	0	1	1	1	1	1	1	1	1	8
Ascherio et al.,1996	0	1	1	1	1	1	1	1	1	8
Esrey et al.,1996	0	0	1	1	1	1	1	1	1	7
Gillman et al.,1997	1	1	1	1	1	1	1	0	1	8
Hu et al.,1997	0	1	1	1	1	1	1	1	1	8
Seino et al.,1997	1	1	1	1	1	1	0	0	1	7
Pietinen et al.,1997	0	1	1	1	1	1	0	1	1	7

Mann et al.,1997	1	1	1	1	1	1	1	0	1	8
Payette et al.,1999	0	1	1	1	1	1	0	0	1	6
Holmes et al.,1999	0	1	1	1	1	1	1	1	1	8
Hu et al.,1999	0	1	1	1	1	1	1	1	1	8
Hu et al.,1999	0	1	1	1	1	1	1	1	1	8
Palli et al.,2000	0	1	1	1	1	1	1	0	1	7
Liu et al.,2000	0	1	1	1	1	1	1	1	1	8
Iso et al.,2001	0	1	1	1	1	1	1	1	1	8
Boniface et al.,2002	1	1	1	1	1	1	0	0	0	6
Hu et al.,2003	0	1	1	1	1	1	1	1	1	8
Zhang et al.,2003	0	1	1	1	1	1	0	1	1	7
He et al.,2003	0	1	1	1	1	1	1	1	1	8
Iso et al.,2003	0	1	1	1	1	1	1	1	1	8
Sauvaget et al.,2004	1	1	1	1	1	1	0	0	1	7
Borugian et al.,2004	0	1	1	1	1	1	0	0	1	6
Jakobsen et al.,2004	1	1	1	1	1	1	0	1	1	8
Tanasescu et al.,2004	0	1	1	1	1	1	1	1	1	8
Kelemen et al.,2005	0	1	1	1	1	1	0	1	1	7
Solfrizzi et al.,2005	1	1	1	1	1	1	1	1	1	9

Leosdottir et al.,2005	1	1	1	1	1	1	1	1	1	9
Tucker et al.,2005	1	1	1	1	1	1	0	1	1	8
Oh et al.,2005	0	1	1	1	1	1	1	1	1	8
Xu et al.,2006	1	1	1	1	1	1	0	1	1	8
Trichopoulou et al. 2006	1	1	1	1	1	1	1	1	1	9
Smit et al.,2007	1	1	1	1	1	1	0	1	1	8
Lagiou et al.,2007	0	1	1	1	1	1	1	1	1	8
Trichopoulou et al. 2007	1	1	1	1	1	1	1	1	1	9
Leosdottir et al.,2007	0	1	1	1	1	1	0	0	1	6
Halbesma et al.,2009	0	1	1	1	1	1	0	0	1	6
Boden-Albala et al.,2009	1	1	1	1	1	1	0	0	1	7
Bates et al.,2010	1	1	1	1	1	1	1	1	1	9
Preis et al.,2010	0	1	1	1	1	1	1	1	1	8
Yamagishi et al.,2010	1	1	1	1	1	1	1	1	1	9
Fung et al.,2010	0	1	1	1	1	1	1	1	1	8
Houston et al.,2011	1	1	1	1	1	1	0	1	1	8
Atkinson et al.,2011	1	1	1	1	1	1	0	0	1	7
Nilsson et al.,2012	1	1	1	1	1	1	0	1	1	8
Dilis et al.,2012	1	1	1	1	1	1	0	1	1	8

Nagata et al.,2012	1	1	1	1	1	1	0	1	1	8
Chiuve et al.,2012	0	1	1	1	1	1	1	1	1	8
Nilsson et al.,2012	1	1	1	1	1	1	0	1	1	8
Wallstrom et al.,2012	1	1	1	1	1	1	0	1	1	8
Larsson et al.,2012	1	1	1	1	1	1	0	1	1	8
Yaemsiri et al.,2012	0	1	1	1	1	1	0	1	1	7
de Oliveira et al.,2012	1	1	1	1	1	1	0	1	1	8
Argos et al.,2013	1	1	1	1	1	1	0	0	1	7
Yamagishi et al.,2013	1	1	1	1	1	1	0	1	1	8
Simila et al.,2013	0	1	1	1	1	1	0	1	1	7
Yu et al.,2013	1	1	1	1	1	1	0	1	1	8
Levine et al.,2014	1	1	1	1	1	1	0	1	1	8
Wakai et al.,2014	1	1	1	1	1	1	0	1	1	8
Miyagawa et al.,2014	1	1	1	1	1	1	0	1	1	8
Rebello et al.,2014	1	1	1	1	1	1	0	1	1	8
Haring et al.,2014	0	1	1	1	1	1	0	1	1	7
Virtanen et al.,2014	0	1	1	1	1	1	0	1	1	7
Santos et al.,2014	0	1	1	1	1	1	0	0	1	6
Nagata et al.,2015	1	1	1	1	1	1	0	1	1	8

Campmans-Kuijpers et al.,2015	0	1	1	1	1	1	0	1	1	7
Puaschitz et al.,2015	0	1	1	1	1	1	1	0	1	7
Chiuve et al.,2015	0	1	1	1	1	1	1	1	1	8
Li et al.,2015	0	1	1	1	1	1	1	1	1	8
Guasch-Ferré et al.,2015	1	1	1	1	1	1	1	1	1	9
Courand et al.,2016	0	1	1	1	1	1	0	0	1	6
Hernández-Alonso et al.,2016	1	1	1	1	1	1	0	1	1	8
Song et al.,2016	0	1	1	1	1	1	1	1	1	8
Owen et al.,2016	1	1	1	1	1	1	0	1	1	8
Wang et al.,2016	0	1	1	1	1	1	1	1	1	8
Xu et al.,2016	0	1	1	1	1	1	0	1	1	7
Chen et al.,2016	0	1	1	1	1	1	1	1	1	8
Praagman et al.,2016	0	1	1	1	1	1	0	1	1	7
Zong et al.,2016	0	1	1	1	1	1	1	1	1	8
Holmes et al.,2017	0	1	1	1	1	1	1	0	1	7
Zaslavsky et al.,2017	0	1	1	1	1	1	0	1	1	7
Dehghan et al.,2017	1	1	1	1	1	1	0	1	1	8
Rhee et al.,2017	0	1	1	1	1	1	0	1	1	7

Arthur et al.,2018	0	1	1	1	1	1	0	1	1	7
Tharrey et al.,2018	1	1	1	1	1	1	0	1	1	8
Song et al.,2018	0	1	1	1	1	1	1	1	1	8
Ricci et al.,2018	1	1	1	1	1	1	1	0	1	8
Seidelmann et al.,2018	1	1	1	1	1	1	0	1	1	8
AlEssa et al.,2018	0	1	1	1	1	1	1	1	1	8
Mazidi et al.,2019	0	1	1	1	1	1	0	1	1	7
Papanikolaou et al.,2019	0	1	1	1	1	1	0	1	1	7
Kurihara et al.,2019	1	1	1	1	1	1	0	1	1	8
Virtanen et al.,2019	0	1	1	1	1	1	0	1	1	7
Budhathoki et al.,2019	1	1	1	1	1	1	0	1	1	8
Chan et al.,2019	0	1	1	1	1	1	0	1	1	7
Jiao et al.,2019	0	1	1	1	1	1	1	1	1	8
Zhuang et al.,2019	1	1	1	1	1	1	0	1	1	8
Zhuang et al.,2019	1	1	1	1	1	1	0	0	1	7
Zhuang et al.,2019	1	1	1	1	1	1	0	1	1	8
Okada et al.,2019	0	1	1	1	1	1	0	1	1	7
Mendonça et al.,2020	0	1	1	1	1	1	0	1	1	7
Ho et al.,2020	1	1	1	1	1	1	1	1	1	9

Langsetmo et al.,2020	0	1	1	1	1	1	0	1	1	7
Chen et al.,2020	0	1	1	1	1	1	0	1	1	7
Huang et al.,2020	1	1	1	1	1	1	0	1	1	8
Trevisan et al.,2020	0	1	1	1	1	1	0	0	1	6
Mao et al.,2020	1	1	1	1	1	1	0	1	1	8
Lelli et al.,2020	0	1	1	1	1	1	0	1	1	7
Ho et al.,2020	1	1	1	1	1	1	1	1	1	9
Mazidi et al.,2020	1	1	1	1	1	1	1	0	1	8
Shan et al.,2020	1	1	1	1	1	1	1	0	1	8
Miyazawa et al.,2020	1	1	1	1	1	1	0	1	1	8
Lin et al.,2020	0	1	1	1	1	1	0	0	1	6
Mirmiran et al.,2020	1	1	1	1	1	1	0	1	1	8
Kwon et al.,2021	1	1	1	1	1	1	0	1	1	8
Yao et al.,2021	0	1	1	1	1	1	0	1	1	7
Akter et al.,2021	1	1	1	1	1	1	0	1	1	8
Sadeghi et al.,2021	1	1	1	1	1	1	0	1	1	8
Sun et al.,2021	0	1	1	1	1	1	0	1	1	7

[a]Maximum 4 points awarded for cohort representativeness, selection of non-exposed cohort, exposure assessment, and demonstration that the outcome is not present at baseline

[b]Maximum 3 points awarded for follow-up length, adequacy of follow-up, and outcome assessment

[c]Maximum 2 points awarded for controlling for the pre-specified primary confounding variable (energy intake) and 3 of the 5 secondary (age,sex,physical activity, smoking status, baseline BMI or body weight) confounding variables

[d]A maximum of 9 points could be awarded. Cohorts with NOS ≥6 are considered high quality.
Abbreviations: BMI = body mass index; NOS = Newcastle-Ottawa Scale.

Table S9. gender subgroup-analysis of dietary macronutrient intake and on mortality or CVD events

Exposure	Endpoint	Stratum	Number of cohorts	Pooled RR(95%CI)	Text of heterogeneity	
					*I*²(%)	*p* value
Total protein	Total mortality	all female	5	0.819(0.661, 1.015)	73.5%	0.004
		female/male≥1	12	0.994(0.909, 1.086)	77.2%	0.000
		female/male<1	3	0.908(0.669, 1.232)	0.0%	0.462
		all male	5	1.005(0.879,1.148)	56.1%	0.058
Total protein	Cancer mortality	all female	6	0.890(0.741, 1.067)	58.6%	0.084
		female/male≥1	7	1.042(0.867, 1.251)	67.3%	0.005
		female/male<1	1	1.000(0.754,1.327)	.	.
		all male	3	0.777(0.544, 1.110)	78.6%	0.009
Total protein	CVD events mortality	all female	4	0.979(0.927, 1.034)	0.0%	0.903
		female/male≥1	8	0.914(0.745,1.121)	59.6%	0.015
		all male	5	0.990(0961, 1.020)	0.0%	0.888
Animal protein	Total mortality	all female	3	0.984(0.939, 1.030)	0.3%	0.367
		female/male≥1	4	1.112(0.979, 1.265)	81.4%	0.001
		all male	3	1.046(0.783,1.398)	69.0%	0.040
Animal protein	Cancer mortality	all female	4	0.960(0.887,1.040)	1.7%	0.384
		female/male≥1	4	1.015(0.921, 1.119)	25.2%	0.260
		female/male<1	1	1.090(0.828, 1.436)	.	.
		all male	1	0.700(0.439,1.117)	.	.
Animal protein	CVD events mortality	all female	4	1.053(0.965,1.149)	0.0%	0.812
		female/male≥1	5	1.094(0.866, 1.381)	75.3%	0.003
		all male	3	1.039(0.874,1.235)	0.0%	0.501
Plant protein	Total mortality	all female	3	0.896(0.801,1.003)	46.3%	0.155
		female/male≥1	4	0.947(0.848, 1.058)	66.9%	0.029
		all male	3	0.953(0.772,1.177)	28.3%	0.248
Plant protein	Cancer mortality	all female	4	0.969(0.809, 1.160)	51.8%	0.101
		female/male≥1	4	0.956(0.875,1.044)	0.0%	0.430
		female/male<1	1	0.820(0.618,1.087)	.	.
		all male	1	0.977(0.897, 1.064)	.	.
Plant protein	CVD events mortality	all female	4	**0.859 (0.781,0.945)**	0.0%	0.602
		female/male≥1	6	0.877 (0.748, 1.028)	29.1%	0.177
		all male	3	0.846 (0.584, 1.227)	40.8%	0.133
Total protein	CVD events	all female	6	**0.816 (0.715, 0.930)**	37.4%	0.157
		female/male≥1	4	0.941 (0.860, 1.029)	0.0%	0.625
		female/male<1	1	1.120 (0.350, 3.620)	.	.
		all male	4	0.902 (0.780, 1.043)	9.3%	0.347
Total fat	CVD events mortality	all female	6	1.025 (0.859, 1.225)	61.5%	0.023
		female/male≥1	10	0.959 (0.876, 1.049)	73.7%	0.000
		all male	5	0.941 (0.788, 1.124)	74.3%	0.004
Total fat	Total mortality	all female	3	1.008 (0.752, 1.351)	89.0%	0.000
		female/male≥1	8	**0.918 (0.827, 0.986)**	81.9%	0.000
		female/male<1	1	0.670 (0.377, 1.191)	.	.
		all male	4	**0.883 (0.783, 0.995)**	46.6%	0.131
SFA	Total mortality	all female	4	1.124 (0.962, 1.313)	51.1%	0.105
		female/male≥1	10	1.074 (0.944, 1.222)	97.0%	0.000
		female/male<1	2	1.016 (0.708, 1.457)	0.0%	0.979

		all male	6	1.015 (0.952, 1.081)	37.1%	0.159
SFA	CVD events	all female	6	1.227 (1.073, 1.403)	14.7%	0.320
	mortality	female/male≥1	11	1.030 (0.962, 1.103)	72.4%	0.000
		all male	7	0.974 (0.897, 1.058)	31.5%	0.188
MUFA	Total	all female	3	1.014 (0.753, 1.366)	82.1%	0.004
	mortality	female/male≥1	9	0.941 (0.870, 1.018)	82.6%	0.000
		female/male<1	2	0.909 (0.705, 1.171)	30.2%	0.231
		all male	4	0.822 (0.761, 0.888)	6.7%	0.360
MUFA	CVD events	all female	5	**0.948 (0.771, 1.165)**	44.0%	0.129
	mortality	female/male≥1	10	**0.935 (0.874, 1.000)**	29.5%	0.173
		all male	5	**0.820 (0.744, 0.903)**	0.0%	0.609
PUFA	Total	all female	3	**0.866(0.811, 0.924)**	0.0%	0.722
	mortality	female/male≥1	10	0.926(0.828, 1.036)	88.8%	0.000
		female/male<1	2	0.839(0.572, 1.230)	50.7%	0.154
		all male	4	**0.866(0.811, 0.924)**	19.4%	0.293
PUFA	CVD events	all female	5	**0.855(0.731, 0.999)**	34.5%	0.191
	mortality	female/male≥1	11	**0.846(0.754, 0.950)**	83.7%	0.000
		all male	5	1.062(0.920, 1.225)	29.1%	0.228
Total fat	CVD events	all female	17	0.983(0.941, 1.026)	0.0%	0.548
		female/male≥1	8	0.899(0.719, 1.125)	60.4%	0.014
		female/male<1	1	1.260(0.953, 1.013)	.	.
		all male	14	0.977(0.925, 1.031)	0.0%	0.723
SFA	CVD events	all female	15	1.048(0.972, 1.131)	3.6%	0.412
		female/male≥1	10	0.901(0.790, 1.027)	59.2%	0.009
		female/male<1	5	0.922(0.673, 1.261)	45.9%	0.116
		all male	19	**0.935(0.874, 0.999)**	0.0%	0.846
MUFA	CVD events	all female	15	0.978(0.883, 1.083)	45.3%	0.029
		female/male≥1	7	0.786(0.585, 1.058)	69.0%	0.004
		female/male<1	2	0.546(0.113, 2.627)	77.5%	0.035
		all male	10	0.927(0.848, 1.012)	17.8%	0.279
PUFA	CVD events	all female	15	0.942(0.876, 1.013)	22.4%	0.205
		female/male≥1	8	0.908(0.749, 1.101)	50.2%	0.050
		female/male<1	2	1.106(0.752, 1.626)	0.0%	0.659
PUFA	CVD events	all male	11	1.030(0.962, 1.104)	0.0%	0.472
Total fat	Stroke	all female	7	0.986(0.917, 1.061)	0.0%	0.638
		female/male≥1	4	0.921(0.595,1.426)	65.9%	0.032
		all male	6	0.952(0.840, 1.079)	0.0%	0.927
SFA	Stroke	all female	6	1.107(0.974, 1.257)	0.0%	0.665
		female/male≥1	7	**0.747(0.664, 0.840)**	14.7%	0.318
		female/male<1	1	**0.220(0.061, 0.798)**	.	.
		all male	9	0.998(0.862, 1.156)	0.0%	0.664
MUFA	Stroke	all female	6	1.032(0.898, 1.186)	0.0%	0.788
		female/male≥1	3	0.714(0.462, 1.102)	26.3%	0.257
		female/male<1	1	0.210(0.051, 0.871)	.	.
		all male	4	0.950(0.771, 1.169)	0.0%	0.990
PUFA	Stroke	all female	6	0.932(0.837, 1.037)	0.0%	0.658
		female/male≥1	3	0.927(0.789, 1.089)	0.0%	0.649
		female/male<1	1	1.390(0.469, 4.120)	.	.
		all male	4	0.961(0.796, 1.160)	0.0%	0.854
Carbohydrates	Total	all female	2	1.004(0.894, 1.128)	86.4%	0.000
	mortality	female/male≥1	6	1.081(0.969, 1.205)	78.3%	0.000

		female/male<1	2	1.688(0.948, 3.007)	45.9%	0.174
		all male	2	1.073(0.888, 1.298)	92.3%	0.000
Carbohydrates	CVD events	all female	9	**1.123(1.045, 1.207)**	0.0%	0.435
		female/male≥1	4	1.008(0.911, 1.115)	72.1%	0.013
		female/male<1	1	0.590(0.220, 1.581)	.	.
		all male	6	1.042(0.984, 1.103)	0.0%	0.809
Carbohydrates	CVD events	all female	4	0.966(0.896, 1.041)	0.0%	0.927
	mortality	female/male≥1	3	1.162(0.886, 1.526)	83.0%	0.003
		all male	4	1.004(0.888, 1.134)	52.6%	0.097

Table S10. religion subgroup-analysis of dietary macronutrient intake and on mortality or CVD events

Exposure	Endpoint	Stratum	Number of cohorts	Pooled RR(95%CI)	Text of heterogeneity	
					*I*²(%)	*p* value
Total protein	Total mortality	North America	9	0.899(0.769, 1.052)	60.8%	0.009
		Europe	11	1.003(0.948, 1.060)	75.2%	0.000
		Asia	4	0.904(0.759, 1.075)	59.1%	0.062
Total protein	Cancer mortality	North America	8	0.836(0.649, 1.076)	73.5%	0.000
		Europe	5	0.989(0.967, 1.012)	0.0%	0.570
		Asia	4	0.922(0.633, 1.344)	66.6%	0.030
Total protein	CVD events mortality	North America	5	0.968(0.825,1.135)	0.0%	0.052
		Europe	5	1.000(0.931, 1.074)	57.5%	0.619
		Asia	6	0.920(0.779,1.086)	17.1%	0.303
Animal protein	Total mortality	North America	3	1.005(0.961, 1.051)	31.2%	0.234
		Europe	4	**1.270(1.071, 1.507)**	55.3%	0.081
		Asia	3	0.921(0.806, 1.053)	22.4%	0.276
Animal protein	Cancer mortality	North America	4	0.987(0.927, 1.051)	11.0%	0.338
		Europe	3	1.113(0.869, 1.424)	44.0%	0.167
		Asia	3	0.918(0.780, 1.081)	4.5%	0.351
Animal protein	CVD events mortality	North America	4	**1.070(1.004, 1.139)**	0.888	0.0%
		Europe	2	1.790(0.796, 4.026)	0.042	75.9%
		Asia	6	0.937(0.766, 1.145)	0.219	28.7%
Plant protein	Total mortality	North America	3	**0.905(0.869,0.943)**	0.0%	0.705
		Europe	4	1.016(0.856,1.206)	37.2%	0.189
		Asia	3	0.869(0.702,1.075)	58.9%	0.088
Plant protein	Cancer mortality	North America	4	0.989(0.889, 1.099)	42.8%	0.155
		Europe	3	0.893(0.741, 1.075)	0.0%	0.375
		Asia	3	0.969(0.689,1.363)	56.2%	0.102
Plant protein	CVD events mortality	North America	4	**0.825(0.741,0.919)**	32.5%	0.217
		Europe	2	1.147(0.884, 1.489)	0.0%	0.371
		Asia	7	**0.811(0.698, 0.942)**	0.0%	0.652
Total protein	CVD events	North America	3	**0.881(0.680, 1.141)**	80.3%	0.006
		Europe	8	**0.855(0.778,0.941)**	11.0%	0.345
Total fat	Total mortality	North America	4	**0.855(0.791, 0.923)**	65.6%	0.033
		Europe	5	0.971(0.758, 1.243)	78.2%	0.001
		Asia	6	0.963(0.856, 1.083)	78.8%	0.000
Total fat	CVD events mortality	North America	10	0.935(0.852, 1.027)	77.6%	0.000
		Europe	7	0.987(0.858,1.135)	60.9%	0.018
		Asia	5	0.999(0.817, 1.221)	69.4%	0.011

SFA	Total mortality	North America	7	1.036(0.930, 1.153)	98.0%	0.000
		Europe	9	1.083(0.986, 1.190)	4.8%	0.395
		Asia	6	1.061(0.939, 1.199)	70.8%	0.004
SFA	CVD events mortality	North America	13	**1.064(1.011, 1.120)**	66.0%	0.000
		Europe	7	0.979(0.785, 1.222)	61.9%	0.015
		Asia	6	0.943(0.836, 1.063)	39.1%	0.145
MUFA	Total mortality	North America	6	0.914(0.832, 1.005)	96.0%	0.000
		Europe	7	0.887(0.739, 1.064)	46.1%	0.084
		Asia	5	0.956(0.897, 1.020)	13.5%	0.328
MUFA	CVD events mortality	North America	12	**0.891(0.823,0.965)**	57.8%	0.006
		Europe	5	**0.822(0.681, 0.992)**	15.4%	0.316
		Asia	5	0.931(0.836, 1.037)	4.1%	0.383
PUFA	Total mortality	North America	7	**0.889(0.833, 0.949)**	81.5%	0.000
		Europe	8	0.903(0.755, 1.080)	70.0%	0.001
		Asia	5	0.947(0.787, 1.140)	89.1%	0.000
PUFA	CVD events mortality	North America	12	**0.882(0.806, 0.965)**	75.7%	0.000
		Europe	6	1.025(0.875, 1.200)	19.3%	0.288
		Asia	5	0.961(0.675, 1.367)	88.5%	0.000
Total fat	CVD events	North America	20	0.994(0.948, 1.042)	15.6%	0.260
		Europe	17	0.957(0.889, 1.030)	10.0%	0.337
		Asia	2	0.685(0.342, 1.374)	42.2%	0.189
SFA	CVD events	North America	16	1.053(0.978, 1.134)	5.6%	0.389
		**Europe**	**21**	**0.934(0.874, 0.998)**	**11.4%**	**0.310**
		Asia	10	**0.815(0.713, 0.932)**	26.4%	0.201
MUFA	CVD events	North America	13	0.946(0.846, 1.059)	54.4%	0.010
		Europe	17	0.977(0.888, 1.075)	32.0%	0.100
		Asia	3	**0.402(0.212, 0.762)**	0.0%	0.486
PUFA	CVD events	North America	13	0.962(0.883, 1.047)	24.7%	0.194
		Europe	18	0.987(0.923, 1.056)	20.3%	0.212
		Asia	3	1.096(0.646, 1.860)	0.0%	0.605
		South America	1	0.580(0.393, 0.856)	.	.
Total fat	Stroke	North America	8	1.006(0.912, 1.109)	19.8%	0.273
		Europe	6	0.989(0.874, 1.119)	0.0%	0.940
		Asia	2	0.685(0.342 1.374)	42.2%	0.189
SFA	Stroke	North America	4	1.109(0.921, 1.336)	0.0%	0.839
		Europe	7	1.083(0.952, 1.233)	0.0%	0.938
		Asia	11	**0.765(0.654, 0.895)**	39.2%	0.087
MUFA	Stroke	North America	3	1.033(0.848, 1.259)	0.0%	0.592
		Europe	7	0.992(0.861, 1.144)	0.0%	0.936
		Asia	3	0.402(0.212, 0.762)	0.0%	0.486
PUFA	Stroke	North America	3	0.974(0.813, 1.167)	0.0%	0.623
		Europe	7	0.926(0.831, 1.032)	0.0%	0.813
		Asia	3	1.096(0.646, 1.860)	0.0%	0.605
Carbohydrates	Total mortality	North America	5	**1.120(1.006, 1.246)**	74.6%	0.001
		Europe	3	**0.969(0.943,0.996)**	8.9%	0.333
		Asia	4	0.987(0.918, 1.061)	0.0%	0.489
Carbohydrates	CVD events	North America	8	**1.079(1.012, 1.150)**	24.5%	0.233
		Europe	7	1.091(0.984, 1.210)	0.0%	0.756
		Asia	2	1.357(0.288, 6.404)	84.9%	0.010
Carbohydrates	CVD events	North America	3	1.187(0.955, 1.477)	73.1%	0.024

mortality	Europe	2	0.979(0.934, 1.026)	0.0%	0.441
	Asia	5	0.953(0.873, 1.040)	0.0%	0.522

Table S11. Begg's test and Egger's test for publication bias among studies

**Exposure**	**Endpoint**	**Begg's Test Pr > |z|**	**Egger's test Pr > |t|**
Total protein	Total mortality	0.941	0.282
Total protein	Cancer mortality	0.711	0.292
Total protein	CVD events mortality	0.108	0.261
Animal protein	Total mortality	0.371	0.539
Animal protein	Cancer mortality	0.721	0.482
Animal protein	CVD events mortality	1.000	0.810
Plant protein	Total mortality	1.000	0.628
Plant protein	Cancer mortality	0.721	0.562
Plant protein	CVD events mortality	0.592	0.314
Total protein	CVD events	0.913	0.996
Animal protein	CVD events	0.348	0.291
Plant protein	CVD events	0.348	0.439
Total protein	Stroke	0.806	0.464
Total fat	Total mortality	0.822	0.351
Total fat	CVD events mortality	0.958	0.505
Total fat	Cancer mortality	0.244	0.153
SFA	Total mortality	0.778	0.869
SFA	CVD events mortality	0.930	0.125
SFA	Cancer mortality	0.876	0.391
MUFA	Total mortality	0.762	0.334
MUFA	CVD events mortality	0.786	0.967
MUFA	Cancer mortality	0.533	0.315
PUFA	Total mortality	0.721	0.417
PUFA	CVD events mortality	0.712	0.764
PUFA	Cancer mortality	0.755	0.223
Total fat	CVD events	0.914	0.916
SFA	CVD events	0.518	0.166
MUFA	CVD events	0.236	0.969
PUFA	CVD events	0.161	0.183
Total fat	CVD	0.152	0.376
SFA	CVD	0.371	0.350
MUFA	CVD	0.711	0.140
PUFA	CVD	0.621	0.529
Total fat	Stroke	0.773	0.577
SFA	Stroke	0.561	0.900
MUFA	Stroke	0.009	0.002
PUFA	Stroke	0.228	0.279
Carbohydrates	Total mortality	0.161	0.049
Carbohydrates	CVD mortality	0.251	0.088
Carbohydrates	Cancer mortality	0.917	0.358
Carbohydrates	CVD events	0.329	0.317
Carbohydrates	Stroke	0.764	0.847
